# Synthesis and structure of (7a*RS*)-4-chloro-6-(4-methyl­phen­yl)-6,7,7a,8-tetra­hydro-5*H*-indeno­[5,6-*b*]furan-5-one, a fused-ring system arising from a new variant of the IMDAV reaction

**DOI:** 10.1107/S2056989026000629

**Published:** 2026-01-27

**Authors:** Kseniia A. Alekseeva, Atash V. Gurbanov, Mikhail S. Grigoriev, Victoria I. Salakhova, Ekaterina A. Akishina, Mohammed Hadi Al-Douh, Tuncer Hökelek

**Affiliations:** a RUDN University, 6 Miklukho-Maklaya St., Moscow 117198, Russian Federation; b Excellence Center, Baku State University, Z. Khalilov Str. 33, AZ 1148, Baku, Azerbaijan; c Frumkin Institute of Physical Chemistry and Electrochemistry, Russian Academy of Sciences, Leninsky prosp. 31, Build. 4, Moscow 119071, Russian Federation; d Institute of Physical Organic Chemistry, National Academy of Sciences of Belarus, Surganov Str. 13, Minsk 220072, Belarus; e Chemistry Department, Faculty of Science, Hadhramout University, Mukalla, Hadhramout, Yemen; f Hacettepe University, Department of Physics, 06800 Beytepe-Ankara, Türkiye; University of Aberdeen, United Kingdom

**Keywords:** iso­indole, furo[2,3-*f*]iso­indole, IMDAV, structure, noncovalent inter­actions, crystal structure

## Abstract

The asymmetric unit of the title com­pound, C_17_H_14_ClNO_2_, contains two mol­ecules, *a* and *b*, where the hydro­benzene and pyrrole rings are in screw-boat and half-chair conformations, respectively. In the crystal, C—H⋯O and C—H⋯Cl hy­dro­gen bonds link the mol­ecules into two-dimensional networks, enclosing *R*_3_^3^(19), *R*_2_^2^(18) and *R*_2_^2^(14) ring motifs. Hirshfeld surface analysis revealed that the most important contributions to the crystal packing are from H⋯H (41.4 and 41.5% for mol­ecules *a* and *b*, respectively), H⋯C/C⋯H (18.1 and 20.2%), H⋯O/O⋯H (16.0 and 13.4%) and H⋯Cl/Cl⋯H (13.4 and 11.9%) inter­actions.

## Abstract 2

The asymmetric unit of the title com­pound contains two mol­ecules. In the crystal, C—H⋯O and C—H⋯Cl hy­dro­gen bonds link the mol­ecules into two-dimensional networks, enclosing *R*_3_^3^(19), *R*_2_^2^(18) and *R*_2_^2^(14) ring motifs. C—H⋯π inter­actions help to consolidate the packing.

## 1. Chemical context

Iso­indole is one of the key heterocyclic scaffolds widely present in natural products, pharmaceuticals and materials (Heugebaert *et al.*, 2012 ▸; Bailly, 2023 ▸). New synthetic approaches to iso­indole-based com­pounds, as well as their applications, continue to be reported regularly (Neto & Zeni, 2021 ▸; Hammouda & Elattar, 2022 ▸; Maharramov *et al.*, 2011 ▸; Ayoup *et al.*, 2023 ▸). We previously introduced a method for the synthesis of a fused iso­indole framework *via* the intra­molecular Diels–Alder reaction of vinyl­arenes (IMDAV strategy) (Krishna *et al.*, 2022 ▸; Voronov *et al.*, 2018 ▸). During this investigation, it was found that the IMDAV reaction of 3-(2-fur­yl)allyl­amines with bromo­maleic anhydride proceeds with concominant de­hydro­bromination, affording the planar heterocyclic com­pound 5-oxo-4a,5,6,7,7a,8-hexa­hydro-4*H*-furo[2,3-*f*]iso­indole (Alekseeva *et al.*, 2020 ▸; Pronina *et al.*, 2024 ▸). This observation prompted us to explore the reactivity of a broader range of 3-(ar­yl)allyl­amines with halogenated maleic anhydrides. In earlier studies, we demonstrated that the reaction of 3-(2-fur­yl)allyl­amine with di­chloro­maleic anhydride delivers the aromatic fused isoindole derivative 6,7-di­hydro-5*H*-furo[2,3-*f*]isoindol-5-one (Alekseeva *et al.*, 2025 ▸). By contrast, replacing di­bromo­maleic acid anhydride with di­chloro­maleic anhydride does not produce the analogous aromatic product. Although the reaction proceeds through the same sequence of elementary transformations, it terminates after deca­rboxylation and elimination. The resulting title com­pound, (7a*RS*)-4-chloro-6-(4-methyl­phen­yl)-6,7,7a,8-tetra­hydro-5*H*-indeno­[5,6-*b*]furan-5-one (**1**), is resistant to further oxidation under ambient conditions or in the presence of various oxidants (see *Synthesis* section). Herein, we describe the synthesis, structure and Hirshfeld surface analysis of (**1**).
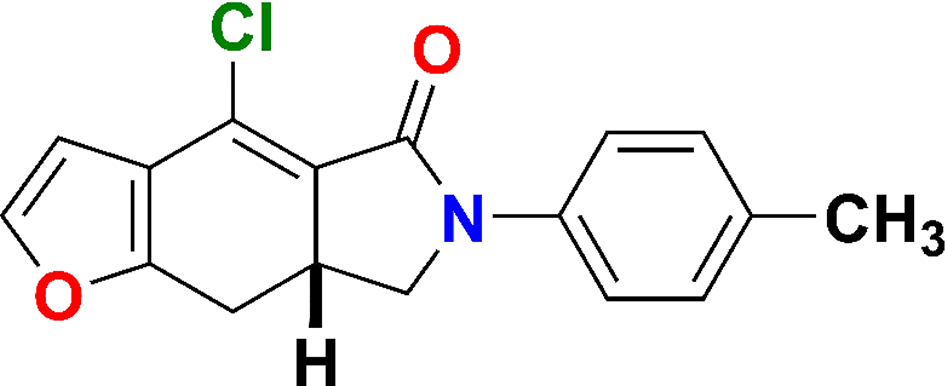


## 2. Structural commentary

**Figure 1:**
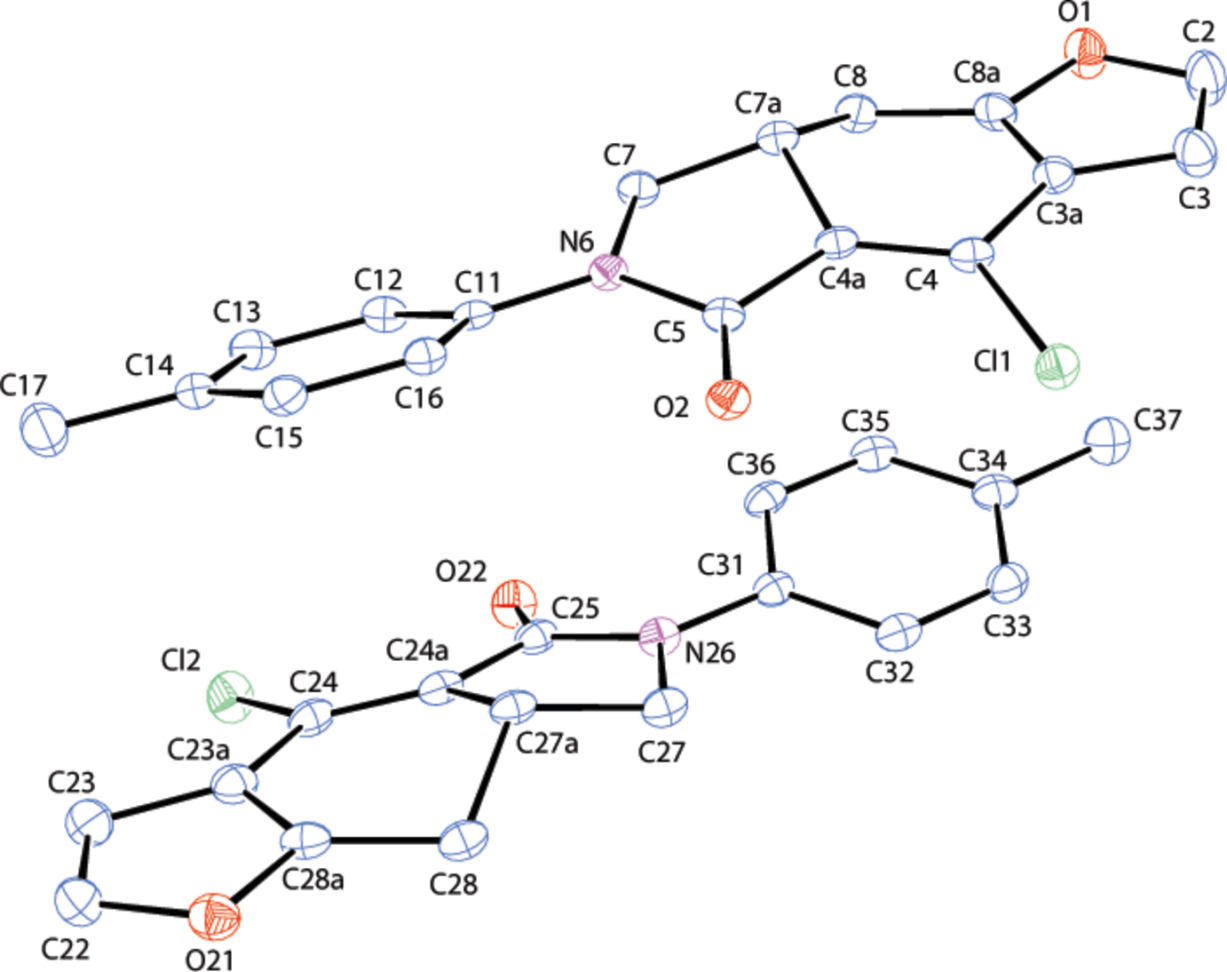
The mol­ecular structure of **1**, shown with 50% probability displacement ellipsoids.

The asymmetric unit of (**1**) contains two crystallographically independent mol­ecules (Fig. 1 ▸), with mol­ecule *a* containing atom N1 and *b* containing N21. The terminal, almost planar, rings *A* (O1/C2/C3/C3*A*/C8*A*) and *D* (C11–C16) in mol­ecule *a*, and the *E* (O21/C22/C23/C23*A*/C28*A*) and *H* (C31–C36) rings in mol­ecule *b* are oriented at dihedral angles of *A*/*D* = 23.26 (6)° and *E*/*H* = 14.62 (6)°. The C atoms of the C17 and C37 methyl groups are displaced by −0.029 (2) and 0.035 (2) Å from their corresponding ring planes. The nonplanar *B* (C3*A*/C4/C4*A*/C7*A*/C8/C8*A*) and *C* (N6/C4*A*/C5/C7/C7*A*) rings in mol­ecule *a* are in screw-boat and half-chair conformations, respectively. The *F* (C23*A*/C24/C24*A*/C27*A*/C28/C28*A*) and *G* (N26/C24*A*/C25/C27/C27*A*) rings in mol­ecule *b* have equivalent conformations. Puckering parameters are *Q*_T_ = 0.3713 (21) Å, θ = 117.15 (31)° and φ = 23.1 (4)° for ring *B*; *Q*_T_ = 0.4054 (21) Å, θ = 63.89 (30)° and φ = 204.3 (3)° for ring *F*; φ = 121.5 (5)° for ring *C* and φ = 308.0 (5)° for ring *G*. In the arbitrarily-chosen asymmetric unit, the stereogenic atoms C7*A* and C27*A* both have *R* configurations, but crystal symmetry generates a racemic mixture.

## 3. Supra­molecular features

**Figure 2:**
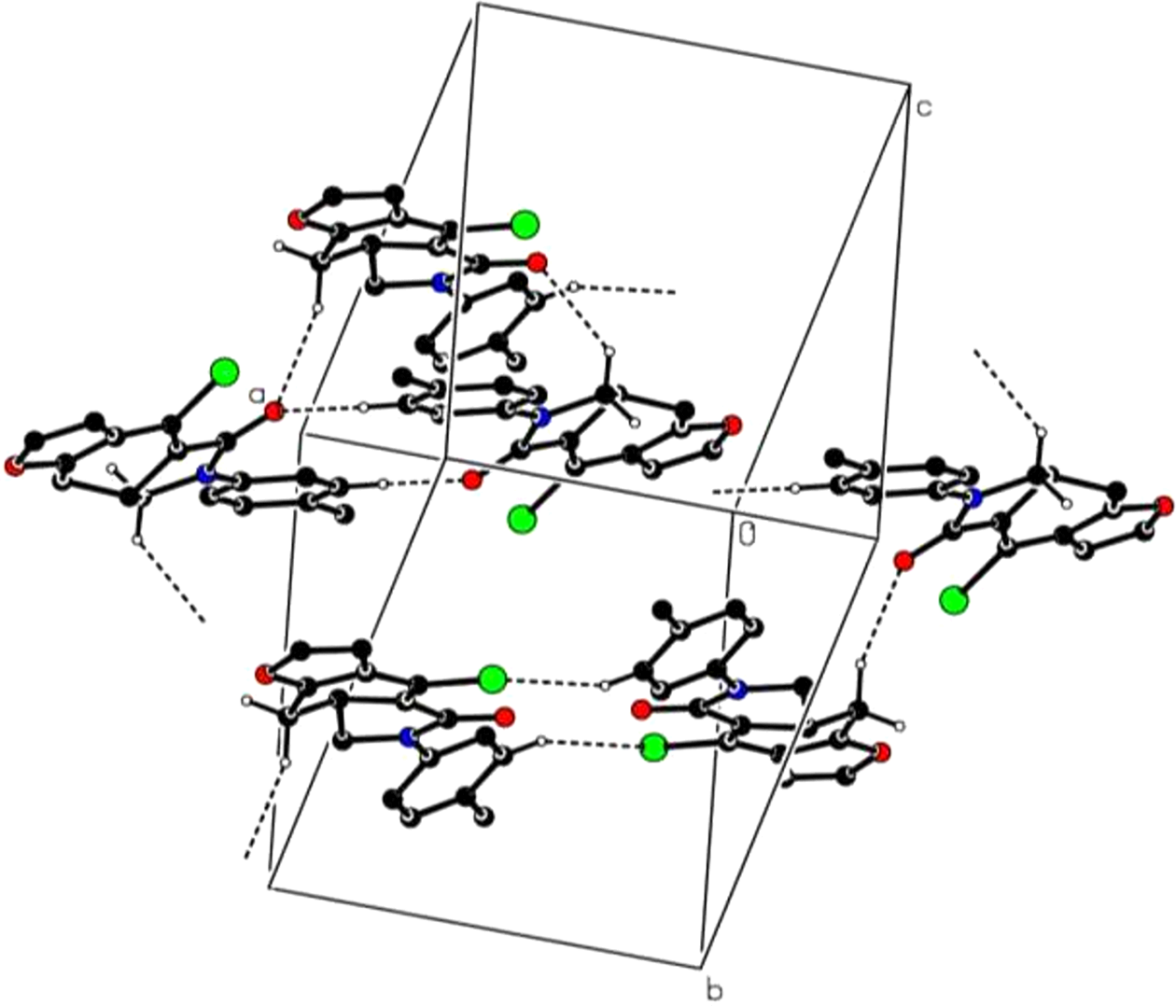
Partial packing diagram of **1**, with C—H⋯O and C—H⋯Cl hy­dro­gen bonds shown as dashed lines. Nonbonding H atoms have been omitted for clarity.

In the crystal, C—H⋯O and C—H⋯Cl hy­dro­gen bonds (Table 1 ▸) link the mol­ecules into two-dimensional networks, enclosing 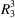
(19), 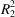
(18) and 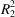
(14) ring motifs (Fig. 2 ▸). Weak C—H⋯π inter­actions help to consolidate the packing.

## 4. Hirshfeld surface analysis

**Figure 3:**
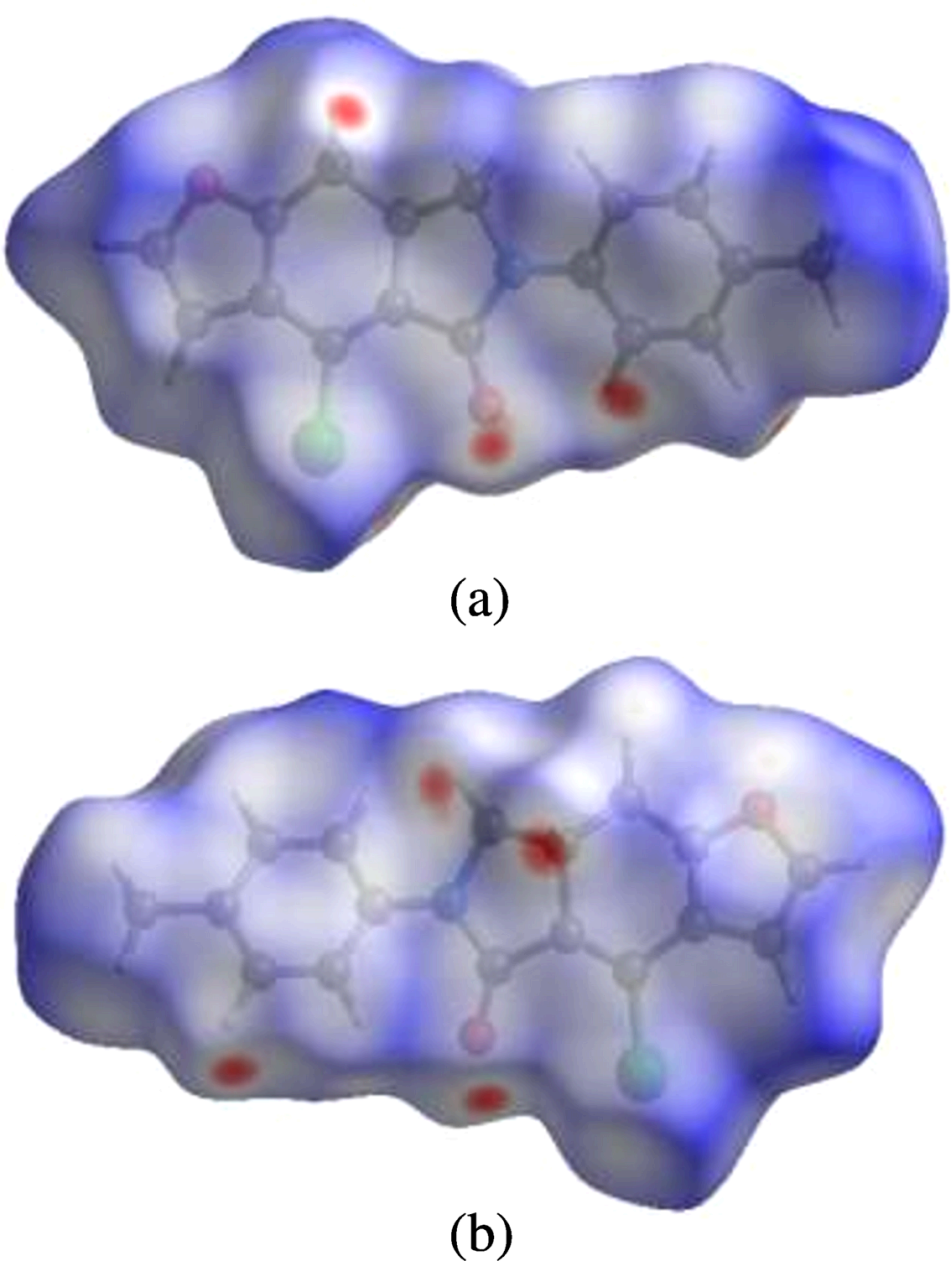
Views of the three-dimensional Hirshfeld surfaces for mol­ecules *a* and *b* plotted over *d*_norm_ in the ranges from −0.16 to 1.36 a.u. and −0.18 to 1.26 a.u., respectively.

**Figure 4:**
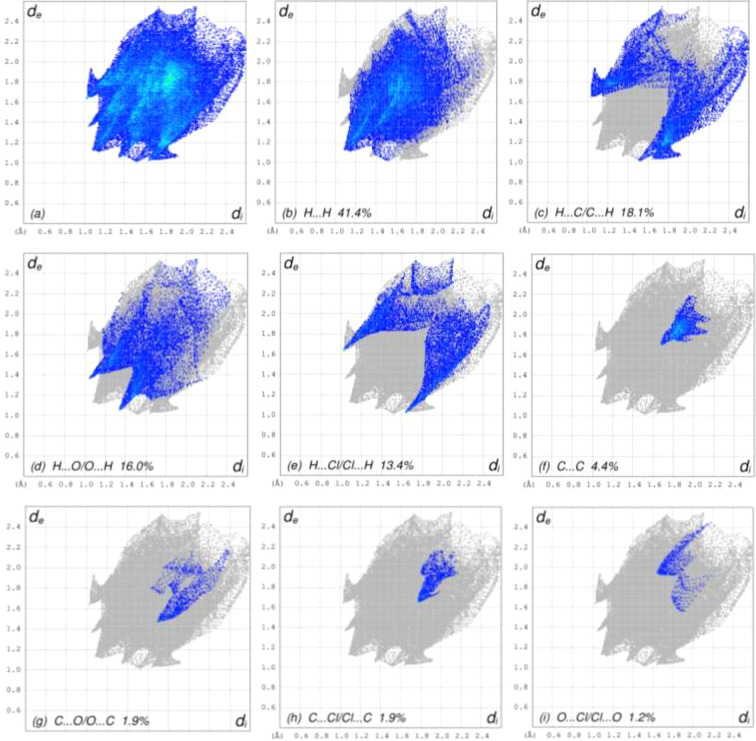
The two-dimensional fingerprint plots for mol­ecule *a*, showing the different inter­action types.

For visualizing the inter­molecular inter­actions in the crystal of (**1**), Hirshfeld surface (HS) analyses were carried out using *CrystalExplorer* (Version 17.5; Spackman *et al.*, 2021 ▸). In the HSs plotted over *d*_norm_ (Fig. 3 ▸), the contact distances equal, shorter and longer with respect to the sum of the van der Waals radii are shown by white, red and blue colours, respectively. According to the two-dimensional fingerprint plots, H⋯H, H⋯C/C⋯H, H⋯O/O⋯H and H⋯Cl/Cl⋯H contacts make the most important contributions to the HSs (Figs. 4 ▸ and 5 ▸, and Table 2 ▸). Slight differences arise in the contact percentages, pre­sum­ably due to the different inter­molecular inter­actions formed by mol­ecules *a* and *b*.

## 5. Synthesis and crystallization

**Figure 6:**
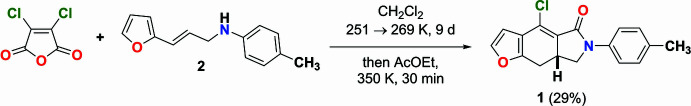
Reaction scheme for obtaining com­pound **1**.

*N*-[(2*E*)-3-(Furan-2-yl)prop-2-en-1-yl]-4-methyl­aniline (0.28 g, 1.3 mmol) (**2**) was dissolved in dry CH_2_Cl_2_ (10 ml) and cooled to 251 K. Di­chloro­maleic anhydride (0.22 g, 1.3 mmol) was added and the mixture was kept at 269 K for 9 d. The resulting precipitate was filtered off, dissolved in AcOEt (10 ml) and stirred at 350 K for 30 min. The precipitate was then filtered off and washed with AcOEt (2 × 2 ml). The product was dried to a constant weight to afford com­pound (**1**) (Fig. 6 ▸) as a white solid (yield: 114.1 mg, 0.38 mmol, 29%; m.p. 425–428 K). A single crystal suitable for X-ray analysis was obtained from DMSO-*d*_6_ solution with heating to 353 K and followed by slow cooling to room tem­per­a­ture. ^1^H NMR (700.2 MHz, DMSO-*d_6_*, 298 K): δ 7.72 (*br dd*, *J* = 1.0, 1.9, 1H, H-2-fur­yl), 7.60 (*d*, *J* = 8.3, 2H, H-2,6-C_6_H_4_), 7.21 (*d*, *J* = 8.4, 2H, H-3,5-C_6_H_4_), 6.70 (*br d*, *J* = 1.9, 1H, H-3-fur­yl), 4.04 (*t*, *J* = 8.8, 1H, H-7A), 3.66 (*dd*, *J* = 7.9, 9.3, 1H, H-7B), 3.54–3.48 (*m*, 1H, H-7A), 3.19 (*dd*, *J* = 9.3, 16.5, 1H, H-8A), 2.82 (*t*, *J* = 16.7, 1H, H-8B), 2.28 (*s*, 3H, CH_3_) ppm. ^13^C NMR (176.1 MHz, DMSO-*d*_6_, 298 K): δ 163.0 (C=O), 154.5, 143.8, 137.2, 133.5, 129.2 (2C, C-2,6-C_6_H_4_), 124.6, 123.0, 120.0, 119.5, (2C, C-3,5-C_6_H_4_), 107.4, 50.8, 35.1, 25.7, 20.5 ppm. IR (KBr), ν (cm^−1^): 3102, 3045, 2840, 2602, 1742, 1694, 1514, 1414, 1253, 836. Analysis calculated (%) for C_17_H_14_ClNO_2_: C 68.12, H 4.71, N 4.67; found: C 67.81, H 4.59, N 4.44.

## 6. Refinement

Crystal data, data collection and structure refinement details are summarized in Table 3 ▸. The H atoms were placed geometrically (C—H = 0.95–1.00 Å) and refined using a riding model, with *U*_iso_(H) = 1.2*U*_eq_(C) or 1.5*U*_eq_(methyl C).

## Supplementary Material

Crystal structure: contains datablock(s) I, global. DOI: 10.1107/S2056989026000629/hb8167sup1.cif

Structure factors: contains datablock(s) I. DOI: 10.1107/S2056989026000629/hb8167Isup2.hkl

Checkcif and printcif files. DOI: 10.1107/S2056989026000629/hb8167sup3.pdf

Supporting information file. DOI: 10.1107/S2056989026000629/hb8167sup4.pdf

CCDC reference: 2525040

Additional supporting information:  crystallographic information; 3D view; checkCIF report

## Figures and Tables

**Figure 5:**
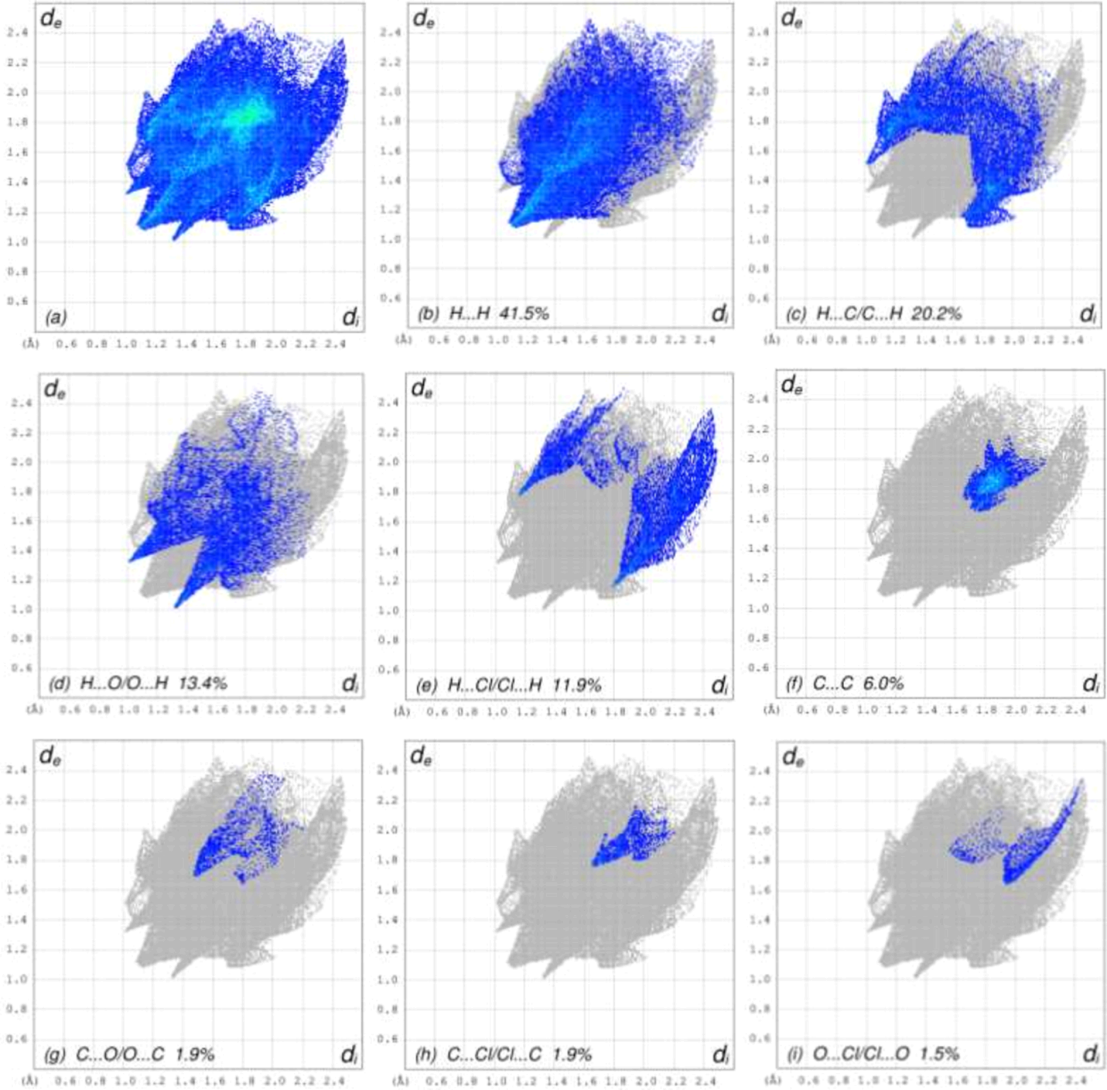
The two-dimensional fingerprint plots for mol­ecule *b*, showing the different inter­action types.

**Table 1:** Hydrogen-bond geometry (Å, °) *Cg*6 and *Cg*8 are the centroids of the C11–C16 and C31–C36 rings, respectively.

*D*—H⋯*A*	*D*—H	H⋯*A*	*D*⋯*A*	*D*—H⋯*A*
C8—H8*A*⋯O22^i^	0.99	2.51	3.401 (2)	149
C15—H15⋯Cl1^ii^	0.95	2.80	3.693 (2)	157
C27—H27*A*⋯O2	0.99	2.48	3.166 (2)	127
C35—H35⋯O22^i^	0.95	2.46	3.252 (2)	141
C28—H28*A*⋯*Cg*8^iii^	0.99	2.72	3.581 (2)	146
C7*A*—H7*AA*⋯*Cg*6^iv^	1.00	2.55	3.484 (2)	155

Symmetry codes: (i) 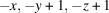; (ii) 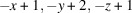; (iii) 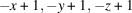; (iv) 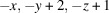.

**Table 2:** Comparison of the atom-type contact percentages for mol­ecules *a* and *b*

Contacts	*a*	*b*
H⋯H	41.4	41.5
H⋯C/C⋯H	18.1	20.2
H⋯O/O⋯H	16.0	13.4
H⋯Cl/Cl⋯H	13.4	11.9
C⋯C	4.4	6.0
C⋯O/O⋯C	1.9	1.9
C⋯Cl/Cl⋯C	1.9	1.9
O⋯Cl/Cl⋯O	1.2	1.5
H⋯N/N⋯H	0.9	1.1
C⋯N/N⋯C	0.3	0.3
N⋯N	0.2	0.1
O⋯O	0.2	0.0
N⋯O/O⋯N	0.2	0.2

**Table 3:** Experimental details

Crystal data	
Chemical formula	C_17_H_14_ClNO_2_
*M*_r_	299.74
Crystal system, space group	Triclinic, *P* 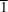
Temperature (K)	100
*a*, *b*, *c* (Å)	9.4921 (7), 10.6159 (8), 15.1129 (11)
α, β, γ (°)	105.490 (3), 104.705 (3), 99.662 (3)
*V* (Å^3^)	1373.39 (18)
*Z*	4
Radiation type	Mo *K*α
μ (mm^−1^)	0.28
Crystal size (mm)	0.40 × 0.32 × 0.28

Data collection	
Diffractometer	Bruker Kappa APEXII area-detector
Absorption correction	Multi-scan (*SADABS2016*; Krause *et al.*, 2015 ▸)
*T*_min_, *T*_max_	0.916, 1.000
No. of measured, independent and observed [*I* > 2σ(*I*)] reflections	24205, 7992, 5466
*R*_int_	0.050
(sin θ/λ)_max_ (Å^−1^)	0.703

Refinement	
*R*[*F*^2^ > 2σ(*F*^2^)], *wR*(*F*^2^), *S*	0.049, 0.114, 1.03
No. of reflections	7992
No. of parameters	381
H-atom treatment	H-atom parameters constrained
Δρ_max_, Δρ_min_ (e Å^−3^)	0.39, −0.38

Computer programs: *APEX3* (Bruker, 2018 ▸), *SAINT* (Bruker, 2018 ▸), *SHELXT* (Sheldrick, 2015*a* ▸), *SHELXL2018* (Sheldrick, 2015*b* ▸), *ORTEP-3 for Windows* (Farrugia, 2012 ▸), *WinGX* (Farrugia, 2012 ▸) and *PLATON* (Spek, 2020 ▸).

## supplementary crystallographic information

### (7aRS)-4-Chloro-6-(4-methylphenyl)-6,7,7a,8-tetrahydro-5H-indeno[5,6-b]furan-5-one Crystal data

**Table:**

C_17_H_14_ClNO_2_	*Z* = 4
*M**_r_* = 299.74	*F*(000) = 624
Triclinic, *P*1	*D*_x_ = 1.450 Mg m^−^^3^
*a* = 9.4921 (7) Å	Mo *K*α radiation, λ = 0.71073 Å
*b* = 10.6159 (8) Å	Cell parameters from 3763 reflections
*c* = 15.1129 (11) Å	θ = 2.9–27.2°
α = 105.490 (3)°	µ = 0.28 mm^−^^1^
β = 104.705 (3)°	*T* = 100 K
γ = 99.662 (3)°	Bulk, colourless
*V* = 1373.39 (18) Å^3^	0.40 × 0.32 × 0.28 mm

### (7aRS)-4-Chloro-6-(4-methylphenyl)-6,7,7a,8-tetrahydro-5H-indeno[5,6-b]furan-5-one Data collection

**Table:**

Bruker Kappa APEXII area-detector diffractometer	5466 reflections with *I* > 2σ(*I*)
φ and ω scans	*R*_int_ = 0.050
Absorption correction: multi-scan (SADABS2016; Krause *et al.*, 2015)	θ_max_ = 30.0°, θ_min_ = 4.1°
*T*_min_ = 0.916, *T*_max_ = 1.000	*h* = −13→13
24205 measured reflections	*k* = −14→13
7992 independent reflections	*l* = −21→21

### (7aRS)-4-Chloro-6-(4-methylphenyl)-6,7,7a,8-tetrahydro-5H-indeno[5,6-b]furan-5-one Refinement

**Table:**

Refinement on *F*^2^	0 restraints
Least-squares matrix: full	Hydrogen site location: inferred from neighbouring sites
*R*[*F*^2^ > 2σ(*F*^2^)] = 0.049	H-atom parameters constrained
*wR*(*F*^2^) = 0.114	*w* = 1/[σ^2^(*F*_o_^2^) + (0.0459*P*)^2^ + 0.1431*P*] where *P* = (*F*_o_^2^ + 2*F*_c_^2^)/3
*S* = 1.03	(Δ/σ)_max_ = 0.001
7992 reflections	Δρ_max_ = 0.39 e Å^−^^3^
381 parameters	Δρ_min_ = −0.38 e Å^−^^3^

### (7aRS)-4-Chloro-6-(4-methylphenyl)-6,7,7a,8-tetrahydro-5H-indeno[5,6-b]furan-5-one Special details

**Table:**

Geometry. All esds (except the esd in the dihedral angle between two l.s. planes) are estimated using the full covariance matrix. The cell esds are taken into account individually in the estimation of esds in distances, angles and torsion angles; correlations between esds in cell parameters are only used when they are defined by crystal symmetry. An approximate (isotropic) treatment of cell esds is used for estimating esds involving l.s. planes.

### (7aRS)-4-Chloro-6-(4-methylphenyl)-6,7,7a,8-tetrahydro-5H-indeno[5,6-b]furan-5-one Fractional atomic coordinates and isotropic or equivalent isotropic displacement parameters (Å^2^)

**Table:**

	*x*	*y*	*z*	*U*_iso_*/*U*_eq_	
Cl1	0.20099 (5)	0.71344 (4)	0.17374 (3)	0.01863 (11)	
O1	−0.34679 (15)	0.54624 (13)	0.08878 (10)	0.0245 (3)	
O2	0.31068 (14)	0.86345 (12)	0.39990 (9)	0.0160 (3)	
N6	0.11862 (16)	0.84991 (13)	0.46830 (11)	0.0135 (3)	
C2	−0.2972 (2)	0.5315 (2)	0.00927 (15)	0.0282 (5)	
H2	−0.359754	0.487656	−0.055801	0.034*	
C3	−0.1489 (2)	0.58738 (19)	0.03607 (14)	0.0231 (4)	
H3	−0.088429	0.589974	−0.005073	0.028*	
C3A	−0.1006 (2)	0.64226 (17)	0.13935 (13)	0.0161 (4)	
C4	0.0447 (2)	0.70905 (16)	0.21219 (13)	0.0146 (4)	
C4A	0.0489 (2)	0.76130 (16)	0.30398 (13)	0.0137 (4)	
C5	0.1779 (2)	0.82930 (16)	0.39256 (13)	0.0132 (3)	
C7	−0.0430 (2)	0.78547 (17)	0.43799 (13)	0.0153 (4)	
H7A	−0.095954	0.844681	0.472261	0.018*	
H7B	−0.060717	0.698063	0.450341	0.018*	
C7A	−0.0948 (2)	0.76485 (17)	0.32964 (13)	0.0151 (4)	
H7AA	−0.124127	0.848108	0.321139	0.018*	
C8	−0.2285 (2)	0.64387 (18)	0.26781 (13)	0.0173 (4)	
H8A	−0.219668	0.564803	0.289777	0.021*	
H8B	−0.324336	0.665991	0.272153	0.021*	
C8A	−0.2236 (2)	0.61474 (17)	0.16722 (14)	0.0176 (4)	
C11	0.20179 (19)	0.91290 (16)	0.56732 (13)	0.0133 (3)	
C12	0.1474 (2)	0.87824 (17)	0.63696 (13)	0.0163 (4)	
H12	0.056975	0.809595	0.617625	0.020*	
C13	0.2239 (2)	0.94284 (18)	0.73415 (14)	0.0189 (4)	
H13	0.184339	0.918209	0.780647	0.023*	
C14	0.3571 (2)	1.04281 (18)	0.76560 (13)	0.0178 (4)	
C15	0.4116 (2)	1.07528 (17)	0.69497 (13)	0.0167 (4)	
H15	0.503493	1.142248	0.714485	0.020*	
C16	0.3360 (2)	1.01299 (16)	0.59750 (13)	0.0143 (4)	
H16	0.375143	1.038167	0.551019	0.017*	
C17	0.4378 (2)	1.1132 (2)	0.87198 (14)	0.0271 (5)	
H17A	0.392484	1.186163	0.896273	0.041*	
H17B	0.544309	1.150899	0.881880	0.041*	
H17C	0.429041	1.048173	0.906968	0.041*	
Cl2	0.45803 (6)	0.71875 (5)	0.81644 (3)	0.02434 (12)	
O21	0.91589 (15)	0.99157 (12)	0.79973 (10)	0.0209 (3)	
O22	0.26448 (15)	0.56575 (12)	0.59949 (9)	0.0213 (3)	
N26	0.37337 (17)	0.61881 (14)	0.48907 (11)	0.0156 (3)	
C22	0.9066 (2)	1.02191 (19)	0.89213 (15)	0.0233 (4)	
H22	0.984685	1.081148	0.948176	0.028*	
C23	0.7725 (2)	0.95676 (18)	0.89282 (14)	0.0226 (4)	
H23	0.739609	0.960197	0.947583	0.027*	
C23A	0.6898 (2)	0.88127 (18)	0.79382 (14)	0.0184 (4)	
C24	0.5468 (2)	0.78260 (17)	0.74464 (14)	0.0171 (4)	
C24A	0.4964 (2)	0.73906 (17)	0.64839 (14)	0.0161 (4)	
C25	0.3642 (2)	0.63252 (17)	0.58064 (14)	0.0161 (4)	
C27	0.5182 (2)	0.69567 (17)	0.49017 (14)	0.0164 (4)	
H27A	0.503727	0.739177	0.439320	0.020*	
H27B	0.586941	0.636469	0.480340	0.020*	
C27A	0.5796 (2)	0.80141 (17)	0.59103 (13)	0.0152 (4)	
H27C	0.546967	0.885038	0.586917	0.018*	
C28	0.7511 (2)	0.84007 (17)	0.63645 (13)	0.0171 (4)	
H28A	0.790695	0.758683	0.625005	0.020*	
H28B	0.799184	0.902961	0.608444	0.020*	
C28A	0.7813 (2)	0.90604 (17)	0.74133 (14)	0.0175 (4)	
C31	0.2638 (2)	0.53323 (16)	0.40167 (13)	0.0146 (4)	
C32	0.3051 (2)	0.49318 (17)	0.31801 (14)	0.0172 (4)	
H32	0.407051	0.520776	0.320751	0.021*	
C33	0.1978 (2)	0.41349 (17)	0.23128 (14)	0.0190 (4)	
H33	0.227942	0.386828	0.175175	0.023*	
C34	0.0470 (2)	0.37116 (17)	0.22377 (14)	0.0181 (4)	
C35	0.0079 (2)	0.41115 (17)	0.30786 (14)	0.0179 (4)	
H35	−0.094066	0.383242	0.305000	0.022*	
C36	0.1134 (2)	0.49030 (17)	0.39536 (14)	0.0168 (4)	
H36	0.083204	0.515619	0.451553	0.020*	
C37	−0.0691 (2)	0.28754 (19)	0.12885 (14)	0.0245 (4)	
H37A	−0.163967	0.254733	0.139807	0.037*	
H37B	−0.034213	0.210403	0.098298	0.037*	
H37C	−0.085093	0.342847	0.086424	0.037*	

### (7aRS)-4-Chloro-6-(4-methylphenyl)-6,7,7a,8-tetrahydro-5H-indeno[5,6-b]furan-5-one Atomic displacement parameters (Å^2^)

**Table:**

	*U*^11^	*U*^22^	*U*^33^	*U*^12^	*U*^13^	*U*^23^
Cl1	0.0133 (2)	0.0242 (2)	0.0188 (2)	0.00305 (17)	0.00713 (19)	0.00671 (18)
O1	0.0135 (7)	0.0344 (8)	0.0186 (7)	0.0008 (6)	0.0019 (6)	0.0035 (6)
O2	0.0102 (6)	0.0176 (6)	0.0185 (7)	0.0018 (5)	0.0046 (5)	0.0042 (5)
N6	0.0102 (7)	0.0145 (7)	0.0159 (8)	0.0020 (6)	0.0053 (6)	0.0047 (6)
C2	0.0209 (11)	0.0404 (12)	0.0168 (11)	0.0036 (9)	0.0031 (9)	0.0039 (9)
C3	0.0171 (10)	0.0310 (10)	0.0180 (10)	0.0031 (8)	0.0036 (8)	0.0065 (8)
C3A	0.0132 (9)	0.0168 (8)	0.0179 (10)	0.0036 (7)	0.0044 (8)	0.0053 (7)
C4	0.0120 (9)	0.0141 (8)	0.0207 (10)	0.0038 (7)	0.0068 (8)	0.0083 (7)
C4A	0.0121 (9)	0.0118 (8)	0.0194 (9)	0.0039 (7)	0.0061 (8)	0.0067 (7)
C5	0.0129 (9)	0.0102 (8)	0.0174 (9)	0.0025 (7)	0.0045 (7)	0.0063 (7)
C7	0.0103 (9)	0.0171 (8)	0.0188 (10)	0.0025 (7)	0.0052 (8)	0.0064 (7)
C7A	0.0127 (9)	0.0140 (8)	0.0195 (10)	0.0043 (7)	0.0054 (8)	0.0060 (7)
C8	0.0101 (9)	0.0199 (9)	0.0203 (10)	0.0016 (7)	0.0043 (8)	0.0057 (7)
C8A	0.0114 (9)	0.0180 (9)	0.0193 (10)	0.0017 (7)	0.0011 (8)	0.0044 (7)
C11	0.0119 (9)	0.0117 (8)	0.0182 (9)	0.0058 (7)	0.0053 (7)	0.0057 (7)
C12	0.0133 (9)	0.0157 (8)	0.0221 (10)	0.0037 (7)	0.0061 (8)	0.0090 (7)
C13	0.0189 (10)	0.0236 (9)	0.0209 (10)	0.0082 (8)	0.0096 (8)	0.0128 (8)
C14	0.0165 (10)	0.0205 (9)	0.0174 (10)	0.0089 (7)	0.0052 (8)	0.0054 (7)
C15	0.0129 (9)	0.0151 (8)	0.0205 (10)	0.0026 (7)	0.0053 (8)	0.0039 (7)
C16	0.0138 (9)	0.0146 (8)	0.0173 (9)	0.0044 (7)	0.0069 (8)	0.0071 (7)
C17	0.0239 (11)	0.0332 (11)	0.0207 (11)	0.0055 (9)	0.0048 (9)	0.0061 (9)
Cl2	0.0249 (3)	0.0303 (3)	0.0217 (3)	0.0036 (2)	0.0120 (2)	0.0122 (2)
O21	0.0180 (7)	0.0202 (6)	0.0238 (8)	0.0042 (5)	0.0056 (6)	0.0072 (6)
O22	0.0213 (7)	0.0225 (7)	0.0229 (8)	0.0018 (6)	0.0112 (6)	0.0102 (6)
N26	0.0152 (8)	0.0150 (7)	0.0192 (8)	0.0032 (6)	0.0078 (7)	0.0079 (6)
C22	0.0243 (11)	0.0246 (10)	0.0193 (10)	0.0081 (8)	0.0036 (9)	0.0065 (8)
C23	0.0242 (11)	0.0249 (10)	0.0203 (10)	0.0085 (8)	0.0069 (9)	0.0086 (8)
C23A	0.0200 (10)	0.0185 (9)	0.0210 (10)	0.0085 (8)	0.0081 (8)	0.0094 (8)
C24	0.0180 (10)	0.0176 (9)	0.0224 (10)	0.0075 (7)	0.0122 (8)	0.0100 (8)
C24A	0.0166 (9)	0.0163 (8)	0.0221 (10)	0.0082 (7)	0.0107 (8)	0.0100 (7)
C25	0.0188 (10)	0.0158 (8)	0.0202 (10)	0.0087 (7)	0.0102 (8)	0.0099 (7)
C27	0.0149 (9)	0.0167 (8)	0.0216 (10)	0.0040 (7)	0.0098 (8)	0.0087 (7)
C27A	0.0150 (9)	0.0149 (8)	0.0217 (10)	0.0068 (7)	0.0097 (8)	0.0101 (7)
C28	0.0161 (9)	0.0154 (8)	0.0238 (10)	0.0050 (7)	0.0103 (8)	0.0082 (7)
C28A	0.0148 (9)	0.0149 (8)	0.0247 (10)	0.0058 (7)	0.0065 (8)	0.0082 (8)
C31	0.0169 (9)	0.0124 (8)	0.0188 (10)	0.0055 (7)	0.0083 (8)	0.0082 (7)
C32	0.0179 (10)	0.0163 (8)	0.0238 (10)	0.0066 (7)	0.0111 (8)	0.0108 (8)
C33	0.0222 (10)	0.0203 (9)	0.0202 (10)	0.0087 (8)	0.0109 (9)	0.0098 (8)
C34	0.0194 (10)	0.0148 (8)	0.0240 (10)	0.0065 (7)	0.0081 (8)	0.0101 (8)
C35	0.0168 (10)	0.0153 (8)	0.0260 (11)	0.0052 (7)	0.0095 (8)	0.0102 (8)
C36	0.0213 (10)	0.0134 (8)	0.0226 (10)	0.0085 (7)	0.0124 (8)	0.0093 (7)
C37	0.0239 (11)	0.0247 (10)	0.0250 (11)	0.0038 (8)	0.0088 (9)	0.0085 (8)

### (7aRS)-4-Chloro-6-(4-methylphenyl)-6,7,7a,8-tetrahydro-5H-indeno[5,6-b]furan-5-one Geometric parameters (Å, º)

**Table:**

Cl1—C4	1.7236 (18)	Cl2—C24	1.7357 (17)
O1—C8A	1.361 (2)	O21—C28A	1.358 (2)
O1—C2	1.379 (2)	O21—C22	1.376 (2)
O2—C5	1.219 (2)	O22—C25	1.224 (2)
N6—C5	1.382 (2)	N26—C25	1.379 (2)
N6—C11	1.414 (2)	N26—C31	1.413 (2)
N6—C7	1.468 (2)	N26—C27	1.470 (2)
C2—C3	1.341 (3)	C22—C23	1.347 (3)
C2—H2	0.9500	C22—H22	0.9500
C3—C3A	1.431 (3)	C23—C23A	1.431 (3)
C3—H3	0.9500	C23—H23	0.9500
C3A—C8A	1.351 (2)	C23A—C28A	1.355 (2)
C3A—C4	1.449 (3)	C23A—C24	1.445 (3)
C4—C4A	1.337 (2)	C24—C24A	1.331 (3)
C4A—C5	1.473 (2)	C24A—C25	1.468 (3)
C4A—C7A	1.514 (2)	C24A—C27A	1.514 (2)
C7—C7A	1.528 (2)	C27—C27A	1.529 (3)
C7—H7A	0.9900	C27—H27A	0.9900
C7—H7B	0.9900	C27—H27B	0.9900
C7A—C8	1.531 (2)	C27A—C28	1.532 (2)
C7A—H7AA	1.0000	C27A—H27C	1.0000
C8—C8A	1.483 (3)	C28—C28A	1.481 (3)
C8—H8A	0.9900	C28—H28A	0.9900
C8—H8B	0.9900	C28—H28B	0.9900
C11—C12	1.389 (2)	C31—C36	1.394 (2)
C11—C16	1.397 (2)	C31—C32	1.398 (2)
C12—C13	1.381 (3)	C32—C33	1.382 (3)
C12—H12	0.9500	C32—H32	0.9500
C13—C14	1.388 (3)	C33—C34	1.392 (3)
C13—H13	0.9500	C33—H33	0.9500
C14—C15	1.394 (2)	C34—C35	1.392 (3)
C14—C17	1.507 (3)	C34—C37	1.500 (3)
C15—C16	1.381 (2)	C35—C36	1.382 (3)
C15—H15	0.9500	C35—H35	0.9500
C16—H16	0.9500	C36—H36	0.9500
C17—H17A	0.9800	C37—H37A	0.9800
C17—H17B	0.9800	C37—H37B	0.9800
C17—H17C	0.9800	C37—H37C	0.9800
			
C8A—O1—C2	105.98 (15)	C28A—O21—C22	105.81 (15)
C5—N6—C11	125.76 (15)	C25—N26—C31	125.57 (15)
C5—N6—C7	113.16 (15)	C25—N26—C27	112.78 (15)
C11—N6—C7	120.76 (14)	C31—N26—C27	121.47 (15)
C3—C2—O1	110.94 (18)	C23—C22—O21	111.23 (18)
C3—C2—H2	124.5	C23—C22—H22	124.4
O1—C2—H2	124.5	O21—C22—H22	124.4
C2—C3—C3A	105.88 (18)	C22—C23—C23A	105.63 (18)
C2—C3—H3	127.1	C22—C23—H23	127.2
C3A—C3—H3	127.1	C23A—C23—H23	127.2
C8A—C3A—C3	106.78 (17)	C28A—C23A—C23	106.58 (17)
C8A—C3A—C4	119.41 (17)	C28A—C23A—C24	118.40 (17)
C3—C3A—C4	133.72 (17)	C23—C23A—C24	134.71 (17)
C4A—C4—C3A	118.35 (16)	C24A—C24—C23A	119.11 (16)
C4A—C4—Cl1	124.56 (14)	C24A—C24—Cl2	123.94 (15)
C3A—C4—Cl1	117.09 (14)	C23A—C24—Cl2	116.76 (14)
C4—C4A—C5	130.45 (17)	C24—C24A—C25	130.58 (16)
C4—C4A—C7A	120.80 (16)	C24—C24A—C27A	120.66 (17)
C5—C4A—C7A	108.64 (15)	C25—C24A—C27A	108.76 (15)
O2—C5—N6	125.69 (17)	O22—C25—N26	125.33 (18)
O2—C5—C4A	128.31 (17)	O22—C25—C24A	127.97 (17)
N6—C5—C4A	105.99 (15)	N26—C25—C24A	106.69 (15)
N6—C7—C7A	103.42 (13)	N26—C27—C27A	103.70 (14)
N6—C7—H7A	111.1	N26—C27—H27A	111.0
C7A—C7—H7A	111.1	C27A—C27—H27A	111.0
N6—C7—H7B	111.1	N26—C27—H27B	111.0
C7A—C7—H7B	111.1	C27A—C27—H27B	111.0
H7A—C7—H7B	109.0	H27A—C27—H27B	109.0
C4A—C7A—C7	102.46 (14)	C24A—C27A—C27	102.94 (14)
C4A—C7A—C8	114.98 (15)	C24A—C27A—C28	113.40 (15)
C7—C7A—C8	115.92 (14)	C27—C27A—C28	115.40 (15)
C4A—C7A—H7AA	107.7	C24A—C27A—H27C	108.3
C7—C7A—H7AA	107.7	C27—C27A—H27C	108.3
C8—C7A—H7AA	107.7	C28—C27A—H27C	108.3
C8A—C8—C7A	105.98 (14)	C28A—C28—C27A	105.98 (15)
C8A—C8—H8A	110.5	C28A—C28—H28A	110.5
C7A—C8—H8A	110.5	C27A—C28—H28A	110.5
C8A—C8—H8B	110.5	C28A—C28—H28B	110.5
C7A—C8—H8B	110.5	C27A—C28—H28B	110.5
H8A—C8—H8B	108.7	H28A—C28—H28B	108.7
C3A—C8A—O1	110.42 (17)	C23A—C28A—O21	110.74 (17)
C3A—C8A—C8	126.87 (17)	C23A—C28A—C28	126.47 (17)
O1—C8A—C8	122.58 (16)	O21—C28A—C28	122.47 (16)
C12—C11—C16	118.72 (17)	C36—C31—C32	118.53 (18)
C12—C11—N6	119.70 (15)	C36—C31—N26	121.87 (16)
C16—C11—N6	121.55 (15)	C32—C31—N26	119.57 (16)
C13—C12—C11	120.48 (17)	C33—C32—C31	120.14 (17)
C13—C12—H12	119.8	C33—C32—H32	119.9
C11—C12—H12	119.8	C31—C32—H32	119.9
C12—C13—C14	121.67 (17)	C32—C33—C34	121.91 (18)
C12—C13—H13	119.2	C32—C33—H33	119.0
C14—C13—H13	119.2	C34—C33—H33	119.0
C13—C14—C15	117.27 (17)	C35—C34—C33	117.24 (18)
C13—C14—C17	120.81 (16)	C35—C34—C37	121.25 (17)
C15—C14—C17	121.92 (17)	C33—C34—C37	121.51 (18)
C16—C15—C14	121.95 (17)	C36—C35—C34	121.81 (18)
C16—C15—H15	119.0	C36—C35—H35	119.1
C14—C15—H15	119.0	C34—C35—H35	119.1
C15—C16—C11	119.90 (16)	C35—C36—C31	120.37 (17)
C15—C16—H16	120.1	C35—C36—H36	119.8
C11—C16—H16	120.1	C31—C36—H36	119.8
C14—C17—H17A	109.5	C34—C37—H37A	109.5
C14—C17—H17B	109.5	C34—C37—H37B	109.5
H17A—C17—H17B	109.5	H37A—C37—H37B	109.5
C14—C17—H17C	109.5	C34—C37—H37C	109.5
H17A—C17—H17C	109.5	H37A—C37—H37C	109.5
H17B—C17—H17C	109.5	H37B—C37—H37C	109.5
			
C8A—O1—C2—C3	0.4 (2)	C28A—O21—C22—C23	0.9 (2)
O1—C2—C3—C3A	−0.4 (2)	O21—C22—C23—C23A	−0.9 (2)
C2—C3—C3A—C8A	0.3 (2)	C22—C23—C23A—C28A	0.5 (2)
C2—C3—C3A—C4	176.56 (19)	C22—C23—C23A—C24	173.68 (19)
C8A—C3A—C4—C4A	−10.9 (2)	C28A—C23A—C24—C24A	−13.0 (3)
C3—C3A—C4—C4A	173.19 (18)	C23—C23A—C24—C24A	174.46 (19)
C8A—C3A—C4—Cl1	168.34 (13)	C28A—C23A—C24—Cl2	162.26 (14)
C3—C3A—C4—Cl1	−7.6 (3)	C23—C23A—C24—Cl2	−10.3 (3)
C3A—C4—C4A—C5	177.46 (15)	C23A—C24—C24A—C25	173.90 (17)
Cl1—C4—C4A—C5	−1.7 (3)	Cl2—C24—C24A—C25	−1.0 (3)
C3A—C4—C4A—C7A	−6.9 (2)	C23A—C24—C24A—C27A	−5.9 (2)
Cl1—C4—C4A—C7A	173.96 (12)	Cl2—C24—C24A—C27A	179.24 (13)
C11—N6—C5—O2	−0.7 (3)	C31—N26—C25—O22	5.3 (3)
C7—N6—C5—O2	−174.11 (15)	C27—N26—C25—O22	−169.96 (17)
C11—N6—C5—C4A	−179.74 (14)	C31—N26—C25—C24A	−175.69 (15)
C7—N6—C5—C4A	6.83 (17)	C27—N26—C25—C24A	9.10 (18)
C4—C4A—C5—O2	7.2 (3)	C24—C24A—C25—O22	5.5 (3)
C7A—C4A—C5—O2	−168.91 (16)	C27A—C24A—C25—O22	−174.75 (17)
C4—C4A—C5—N6	−173.78 (17)	C24—C24A—C25—N26	−173.57 (18)
C7A—C4A—C5—N6	10.12 (17)	C27A—C24A—C25—N26	6.23 (18)
C5—N6—C7—C7A	−20.48 (18)	C25—N26—C27—C27A	−20.27 (18)
C11—N6—C7—C7A	165.72 (13)	C31—N26—C27—C27A	164.30 (14)
C4—C4A—C7A—C7	161.67 (15)	C24—C24A—C27A—C27	161.95 (16)
C5—C4A—C7A—C7	−21.79 (16)	C25—C24A—C27A—C27	−17.86 (18)
C4—C4A—C7A—C8	35.0 (2)	C24—C24A—C27A—C28	36.6 (2)
C5—C4A—C7A—C8	−148.43 (14)	C25—C24A—C27A—C28	−143.25 (15)
N6—C7—C7A—C4A	24.41 (16)	N26—C27—C27A—C24A	21.99 (17)
N6—C7—C7A—C8	150.44 (14)	N26—C27—C27A—C28	146.07 (14)
C4A—C7A—C8—C8A	−40.77 (19)	C24A—C27A—C28—C28A	−43.86 (18)
C7—C7A—C8—C8A	−160.18 (15)	C27—C27A—C28—C28A	−162.26 (13)
C3—C3A—C8A—O1	−0.1 (2)	C23—C23A—C28A—O21	0.0 (2)
C4—C3A—C8A—O1	−176.96 (14)	C24—C23A—C28A—O21	−174.44 (14)
C3—C3A—C8A—C8	175.90 (17)	C23—C23A—C28A—C28	173.59 (16)
C4—C3A—C8A—C8	−1.0 (3)	C24—C23A—C28A—C28	−0.9 (3)
C2—O1—C8A—C3A	−0.2 (2)	C22—O21—C28A—C23A	−0.57 (19)
C2—O1—C8A—C8	−176.36 (17)	C22—O21—C28A—C28	−174.43 (15)
C7A—C8—C8A—C3A	26.7 (2)	C27A—C28—C28A—C23A	29.3 (2)
C7A—C8—C8A—O1	−157.82 (15)	C27A—C28—C28A—O21	−157.86 (15)
C5—N6—C11—C12	−152.02 (16)	C25—N26—C31—C36	23.6 (2)
C7—N6—C11—C12	20.9 (2)	C27—N26—C31—C36	−161.56 (15)
C5—N6—C11—C16	30.0 (2)	C25—N26—C31—C32	−158.39 (16)
C7—N6—C11—C16	−157.00 (15)	C27—N26—C31—C32	16.4 (2)
C16—C11—C12—C13	0.8 (2)	C36—C31—C32—C33	0.4 (2)
N6—C11—C12—C13	−177.24 (15)	N26—C31—C32—C33	−177.65 (14)
C11—C12—C13—C14	−0.6 (3)	C31—C32—C33—C34	0.3 (3)
C12—C13—C14—C15	−0.3 (3)	C32—C33—C34—C35	−0.7 (2)
C12—C13—C14—C17	179.27 (17)	C32—C33—C34—C37	178.48 (15)
C13—C14—C15—C16	1.1 (3)	C33—C34—C35—C36	0.4 (2)
C17—C14—C15—C16	−178.52 (17)	C37—C34—C35—C36	−178.76 (15)
C14—C15—C16—C11	−0.9 (3)	C34—C35—C36—C31	0.3 (2)
C12—C11—C16—C15	0.0 (2)	C32—C31—C36—C35	−0.7 (2)
N6—C11—C16—C15	177.92 (15)	N26—C31—C36—C35	177.33 (14)

### (7aRS)-4-Chloro-6-(4-methylphenyl)-6,7,7a,8-tetrahydro-5H-indeno[5,6-b]furan-5-one Hydrogen-bond geometry (Å, º)

Cg6 and Cg8 are the centroids of the (C11···C16) and (C31···C36), respectively.

**Table:**

*D*—H···*A*	*D*—H	H···*A*	*D*···*A*	*D*—H···*A*
C8—H8*A*···O22^i^	0.99	2.51	3.401 (2)	149
C15—H15···Cl1^ii^	0.95	2.80	3.693 (2)	157
C27—H27*A*···O2	0.99	2.48	3.166 (2)	127
C35—H35···O22^i^	0.95	2.46	3.252 (2)	141
C28—H28*A*···*Cg*8^iii^	0.99	2.72	3.581 (2)	146
C7*A*—H7*AA*···*Cg*6^iv^	1.00	2.55	3.484 (2)	155

Symmetry codes: (i) −*x*, −*y*+1, −*z*+1; (ii) −*x*+1, −*y*+2, −*z*+1; (iii) −*x*+1, −*y*+1, −*z*+1; (iv) −*x*, −*y*+2, −*z*+1.
