# Supplementary material for: Synthesis and structure of (7aRS)-4-chloro-6-(4-methyl­phen­yl)-6,7,7a,8-tetra­hydro-5H-indeno­[5,6-b]furan-5-one, a fused-ring system arising from a new variant of the IMDAV reaction
Source: Acta Crystallogr E Crystallogr Commun. 2026 Jan 27;82(Pt 2):212–6. doi: 10.1107/S2056989026000629 (PMC12874248; doi:10.1107/S2056989026000629)

checkCIF/PLATON report

Structure factors have been supplied for datablock(s) FZ3117_LT

THIS REPORT IS FOR GUIDANCE ONLY. IF USED AS PART OF A REVIEW PROCEDURE FOR PUBLICATION, IT SHOULD NOT REPLACE THE EXPERTISE OF AN EXPERIENCED CRYSTALLOGRAPHIC REFEREE.

No syntax errors found.   CIF dictionary   Interpreting this report

Datablock: FZ3117_LT

```
Bond precision:   C-C = 0.0030 A            Wavelength=0.71073

Cell:          a=9.4921(7)        b=10.6159(8)       c=15.1129(11)
               alpha=105.490(3)   beta=104.705(3)    gamma=99.662(3)
Temperature:   100 K

                Calculated                  Reported
Volume          1373.40(18)                 1373.39(18)
Space group     P -1                        P -1
Hall group      -P 1                        -P 1
Moiety formula  C17 H14 Cl N O2             C17 H14 Cl N O2
Sum formula     C17 H14 Cl N O2             C17 H14 Cl N O2
Mr              299.74                      299.74
Dx,g cm-3       1.450                       1.450
Z               4                           4
Mu (mm-1)       0.282                       0.282
F000            624.0                       624.0
F000'           624.85
h,k,lmax        13,14,21                    13,14,21
Nref            8014                        7992
Tmin,Tmax       0.897,0.924                 0.916,1.000
Tmin'           0.893

Correction method= # Reported T Limits: Tmin=0.916 Tmax=1.000
AbsCorr = MULTI-SCAN
```

Data completeness= 0.997 Theta(max)= 29.998

R(reflections)= 0.0494(5466) wR2(reflections)= 0.1136(7992)

S = 1.033 Npar= 381

The following ALERTS were generated. Each ALERT has the format
test-name_ALERT_alert-type_alert-level.
Click on the hyperlinks for more details of the test.

Alert level B

```
PLAT910_ALERT_3_B Missing FCF Reflection(s) Below Theta(Min)[Deg]=       4.11 Note
                   1  0  0,  -1  1  0,   0  1  0,   1  1  0,   0 -2  1,  -1 -1  1,
                   0 -1  1,   1 -1  1,  -1  0  1,   0  0  1,   1  0  1,  -1  1  1,
                   0  1  1,  -1 -1  2,   0 -1  2,   1 -1  2,  -1  0  2,   0  0  2,
                  -1  1  2,
```

Author Response: Closed by beamstop. The maximum dimension of the crystal was 0.4 mm, and the collimator used was 0.6 mm. The shadow of the beamstop was closing the reflections with resolution of about 5 \%A^-1^. It also depends on the quality (mosaicity) of the crystal. For the crystal, it was only possible to reduce the cutoff to 5.5 \%A^-1^ with 16 reflections missing.

Alert level C

```
PLAT714_ALERT_1_C CONTACT Unknown or Inconsistent Label .........         I1 Check
                    I1          I2
PLAT714_ALERT_1_C CONTACT Unknown or Inconsistent Label .........         I1 Check
                    I1          H10A
PLAT714_ALERT_1_C CONTACT Unknown or Inconsistent Label .........        O10 Check
                    O1          O10
PLAT714_ALERT_1_C CONTACT Unknown or Inconsistent Label .........         O3 Check
                    O3          C6
PLAT714_ALERT_1_C CONTACT Unknown or Inconsistent Label .........         O4 Check
                    O4          O1
PLAT714_ALERT_1_C CONTACT Unknown or Inconsistent Label .........         O4 Check
                    O4          C3A
PLAT714_ALERT_1_C CONTACT Unknown or Inconsistent Label .........         O4 Check
                    O4          O6
PLAT714_ALERT_1_C CONTACT Unknown or Inconsistent Label .........         O4 Check
                    O4          C1
PLAT714_ALERT_1_C CONTACT Unknown or Inconsistent Label .........         O5 Check
                    O5          O11
PLAT714_ALERT_1_C CONTACT Unknown or Inconsistent Label .........         O5 Check
                    O5          O6
PLAT714_ALERT_1_C CONTACT Unknown or Inconsistent Label .........         O6 Check
                    O6          C36
PLAT714_ALERT_1_C CONTACT Unknown or Inconsistent Label .........         O7 Check
                    O7          C28
PLAT714_ALERT_1_C CONTACT Unknown or Inconsistent Label .........         O8 Check
```

```
                   O8          C30
PLAT714_ALERT_1_C CONTACT Unknown or Inconsistent Label .........        O10 Check
                   O10         C27A
PLAT714_ALERT_1_C CONTACT Unknown or Inconsistent Label .........        O10 Check
                   O10         C25
PLAT714_ALERT_1_C CONTACT Unknown or Inconsistent Label .........       H10O Check
                   O1          H10O
PLAT714_ALERT_1_C CONTACT Unknown or Inconsistent Label .........       H49A Check
                   O1          H49A
PLAT714_ALERT_1_C CONTACT Unknown or Inconsistent Label .........       H50B Check
                   O1          H50B
PLAT714_ALERT_1_C CONTACT Unknown or Inconsistent Label .........        H18 Check
                   O2          H18
PLAT714_ALERT_1_C CONTACT Unknown or Inconsistent Label .........        H4B Check
                   O2          H4B
PLAT714_ALERT_1_C CONTACT Unknown or Inconsistent Label .........        H30 Check
                   O2          H30
PLAT714_ALERT_1_C CONTACT Unknown or Inconsistent Label .........         O3 Check
                   O3          H6
PLAT714_ALERT_1_C CONTACT Unknown or Inconsistent Label .........         O3 Check
                   O3          H47B
PLAT714_ALERT_1_C CONTACT Unknown or Inconsistent Label .........         O3 Check
                   O3          H22
PLAT714_ALERT_1_C CONTACT Unknown or Inconsistent Label .........         O4 Check
                   O4          H50C
PLAT714_ALERT_1_C CONTACT Unknown or Inconsistent Label .........         O4 Check
                   O4          H42
PLAT714_ALERT_1_C CONTACT Unknown or Inconsistent Label .........         O4 Check
                   O4          H27B
PLAT714_ALERT_1_C CONTACT Unknown or Inconsistent Label .........         O4 Check
                   O4          H3C
PLAT714_ALERT_1_C CONTACT Unknown or Inconsistent Label .........         O4 Check
                   O4          H49B
PLAT714_ALERT_1_C CONTACT Unknown or Inconsistent Label .........         O5 Check
                   O5          H27B
PLAT714_ALERT_1_C CONTACT Unknown or Inconsistent Label .........         O5 Check
                   O5          H40
PLAT714_ALERT_1_C CONTACT Unknown or Inconsistent Label .........         O5 Check
                   O5          H11O
PLAT714_ALERT_1_C CONTACT Unknown or Inconsistent Label .........         O6 Check
                   O6          H5O
PLAT714_ALERT_1_C CONTACT Unknown or Inconsistent Label .........         O6 Check
                   O6          H36
PLAT714_ALERT_1_C CONTACT Unknown or Inconsistent Label .........         O7 Check
                   O7          H28A
PLAT714_ALERT_1_C CONTACT Unknown or Inconsistent Label .........         O7 Check
                   O7          H3A
PLAT714_ALERT_1_C CONTACT Unknown or Inconsistent Label .........         O7 Check
                   O7          H42
PLAT714_ALERT_1_C CONTACT Unknown or Inconsistent Label .........         O7 Check
                   O7          H28B
PLAT714_ALERT_1_C CONTACT Unknown or Inconsistent Label .........         O8 Check
                   O8          H46
PLAT714_ALERT_1_C CONTACT Unknown or Inconsistent Label .........         O8 Check
                   O8          H30
PLAT714_ALERT_1_C CONTACT Unknown or Inconsistent Label .........         O8 Check
                   O8          H4B
```

```
PLAT714_ALERT_1_C CONTACT Unknown or Inconsistent Label .........          O8 Check
                    O8          H18
PLAT714_ALERT_1_C CONTACT Unknown or Inconsistent Label .........          O9 Check
                    O9          H12
PLAT714_ALERT_1_C CONTACT Unknown or Inconsistent Label .........         O10 Check
                    O10         H27C
PLAT714_ALERT_1_C CONTACT Unknown or Inconsistent Label .........         O10 Check
                    O10         H23C
PLAT714_ALERT_1_C CONTACT Unknown or Inconsistent Label .........         O11 Check
                    O11         H10
PLAT714_ALERT_1_C CONTACT Unknown or Inconsistent Label .........         O11 Check
                    O11         H43
PLAT714_ALERT_1_C CONTACT Unknown or Inconsistent Label .........         C43 Check
                    C12         C43
PLAT714_ALERT_1_C CONTACT Unknown or Inconsistent Label .........         C42 Check
                    C12         C42
PLAT714_ALERT_1_C CONTACT Unknown or Inconsistent Label .........         C21 Check
                    C21         C22
PLAT714_ALERT_1_C CONTACT Unknown or Inconsistent Label .........         C21 Check
                    C21         C40
PLAT714_ALERT_1_C CONTACT Unknown or Inconsistent Label .........         C21 Check
                    C21         C35
PLAT714_ALERT_1_C CONTACT Unknown or Inconsistent Label .........          C1 Check
                    C1          H12
PLAT714_ALERT_1_C CONTACT Unknown or Inconsistent Label .........        H10A Check
                    C4A         H10A
PLAT714_ALERT_1_C CONTACT Unknown or Inconsistent Label .........         H39 Check
                    C7          H39
PLAT714_ALERT_1_C CONTACT Unknown or Inconsistent Label .........         H39 Check
                    C8          H39
PLAT714_ALERT_1_C CONTACT Unknown or Inconsistent Label .........         C10 Check
                    C10         H11O
PLAT714_ALERT_1_C CONTACT Unknown or Inconsistent Label .........         H3A Check
                    C16         H3A
PLAT714_ALERT_1_C CONTACT Unknown or Inconsistent Label .........         C18 Check
                    C18         H27A
PLAT714_ALERT_1_C CONTACT Unknown or Inconsistent Label .........         C19 Check
                    C19         H27A
PLAT714_ALERT_1_C CONTACT Unknown or Inconsistent Label .........         C21 Check
                    C21         H22
PLAT714_ALERT_1_C CONTACT Unknown or Inconsistent Label .........        H50C Check
                    C24         H50C
PLAT714_ALERT_1_C CONTACT Unknown or Inconsistent Label .........        H11O Check
                    C24         H11O
PLAT714_ALERT_1_C CONTACT Unknown or Inconsistent Label .........         H3C Check
                    C24         H3C
PLAT714_ALERT_1_C CONTACT Unknown or Inconsistent Label .........         H5O Check
                    C25         H5O
PLAT714_ALERT_1_C CONTACT Unknown or Inconsistent Label .........         H40 Check
                    C27         H40
PLAT714_ALERT_1_C CONTACT Unknown or Inconsistent Label .........        H34A Check
                    C28A        H34A
PLAT714_ALERT_1_C CONTACT Unknown or Inconsistent Label .........        H34A Check
                    C35         H34A
PLAT714_ALERT_1_C CONTACT Unknown or Inconsistent Label .........         C40 Check
                    C40         H27A
PLAT714_ALERT_1_C CONTACT Unknown or Inconsistent Label .........         C40 Check
```

```
                    C40          H34
PLAT714_ALERT_1_C CONTACT Unknown or Inconsistent Label .........        C43 Check
                    C43          H37
PLAT714_ALERT_1_C CONTACT Unknown or Inconsistent Label .........        H3A Check
                    H3A          H16
PLAT714_ALERT_1_C CONTACT Unknown or Inconsistent Label .........        H10 Check
                    H10          H11O
PLAT714_ALERT_1_C CONTACT Unknown or Inconsistent Label .........       H10O Check
                    H10O         H12
PLAT714_ALERT_1_C CONTACT Unknown or Inconsistent Label .........       H11O Check
                    H11O         H50C
PLAT714_ALERT_1_C CONTACT Unknown or Inconsistent Label .........        H19 Check
                    H15          H19
PLAT714_ALERT_1_C CONTACT Unknown or Inconsistent Label .........        H21 Check
                    H21          H23B
PLAT714_ALERT_1_C CONTACT Unknown or Inconsistent Label .........        H40 Check
                    H27B         H40
PLAT714_ALERT_1_C CONTACT Unknown or Inconsistent Label .........        H42 Check
                    H28A         H42
PLAT714_ALERT_1_C CONTACT Unknown or Inconsistent Label .........        H45 Check
                    H45          H47C
```

Alert level G

```
PLAT154_ALERT_1_G The s.u.'s on the Cell Angles are Equal ..(Note)      0.003 Degree
PLAT398_ALERT_2_G Deviating  C-O-C   Angle From 120 for O1        .      106.0 Degree
PLAT398_ALERT_2_G Deviating  C-O-C   Angle From 120 for O21       .      105.8 Degree
PLAT484_ALERT_4_G Round D-H..A Angle Rep for C8       ..O22       .        149 Degree
PLAT484_ALERT_4_G Round D-H..A Angle Rep for C27      ..O2        .        127 Degree
PLAT720_ALERT_4_G Number of Unusual/Non-Standard Labels .........          1 Note
              H7AA
PLAT793_ALERT_4_G Model has Chirality at C7A         (Centro SpGr)          R Verify
PLAT793_ALERT_4_G Model has Chirality at C27A        (Centro SpGr)          R Verify
PLAT883_ALERT_1_G Absent Datum for _atom_sites_solution_primary ..     Please Do !
PLAT899_ALERT_4_G SHELXL2018 is Outdated   and Succeeded by SHELXL     2019/3 Note
PLAT912_ALERT_4_G Missing # of FCF Reflections Above STh/L=  0.600          3 Note
PLAT941_ALERT_3_G Average HKL Measurement Multiplicity ..........        3.0 Low
PLAT967_ALERT_5_G Note: Two-Theta Cutoff Value in Embedded .res ..       60.0 Degree
PLAT969_ALERT_5_G The 'Henn et al.' R-Factor-gap value ..........      2.252 Note
              Predicted wR2: Based on SigI**2  5.05 or SHELX Weight 11.00
PLAT978_ALERT_2_G Number C-C Bonds with Positive Residual Density.         11 Info
```

---

  0 **ALERT level A** = Most likely a serious problem - resolve or explain
  1 **ALERT level B** = A potentially serious problem, consider carefully
 80 **ALERT level C** = Check. Ensure it is not caused by an omission or oversight
 15 **ALERT level G** = General information/check it is not something unexpected

```
 82 ALERT type 1 CIF construction/syntax error, inconsistent or missing data
  3 ALERT type 2 Indicator that the structure model may be wrong or deficient
  2 ALERT type 3 Indicator that the structure quality may be low
  7 ALERT type 4 Improvement, methodology, query or suggestion
  2 ALERT type 5 Informative message, check
```

Publication of your CIF

You should attempt to resolve as many as possible of the alerts in all categories. Often the minor alerts point to easily fixed oversights, errors and omissions in your CIF or refinement strategy, so attention to these fine details can be worthwhile. In order to resolve some of the more serious problems it may be necessary to carry out additional measurements or structure refinements. However, the nature of your study may justify the reported deviations from journal submission requirements and the more serious of these should be commented upon in the discussion or experimental section of a paper or in the "special_details" fields of the CIF. *checkCIF* was carefully designed to identify outliers and unusual parameters, but every test has its limitations and alerts that are not important in a particular case may appear. Conversely, the absence of alerts does not guarantee there are no aspects of the results needing attention. It is up to the individual to critically assess their own results and, if necessary, seek expert advice.

If you wish to submit your CIF for publication in Acta Crystallographica Section C or E, you should upload your CIF via the web. If you wish to submit your CIF for publication in IUCrData you should upload your CIF via the web. If your CIF is to form part of a submission to another IUCr journal, you will be asked, either during electronic submission or by the Co-editor handling your paper, to upload your CIF via our web site.

PLATON version of 04/06/2025; check.def file version of 30/05/2025

Datablock FZ3117_LT - ellipsoid plot

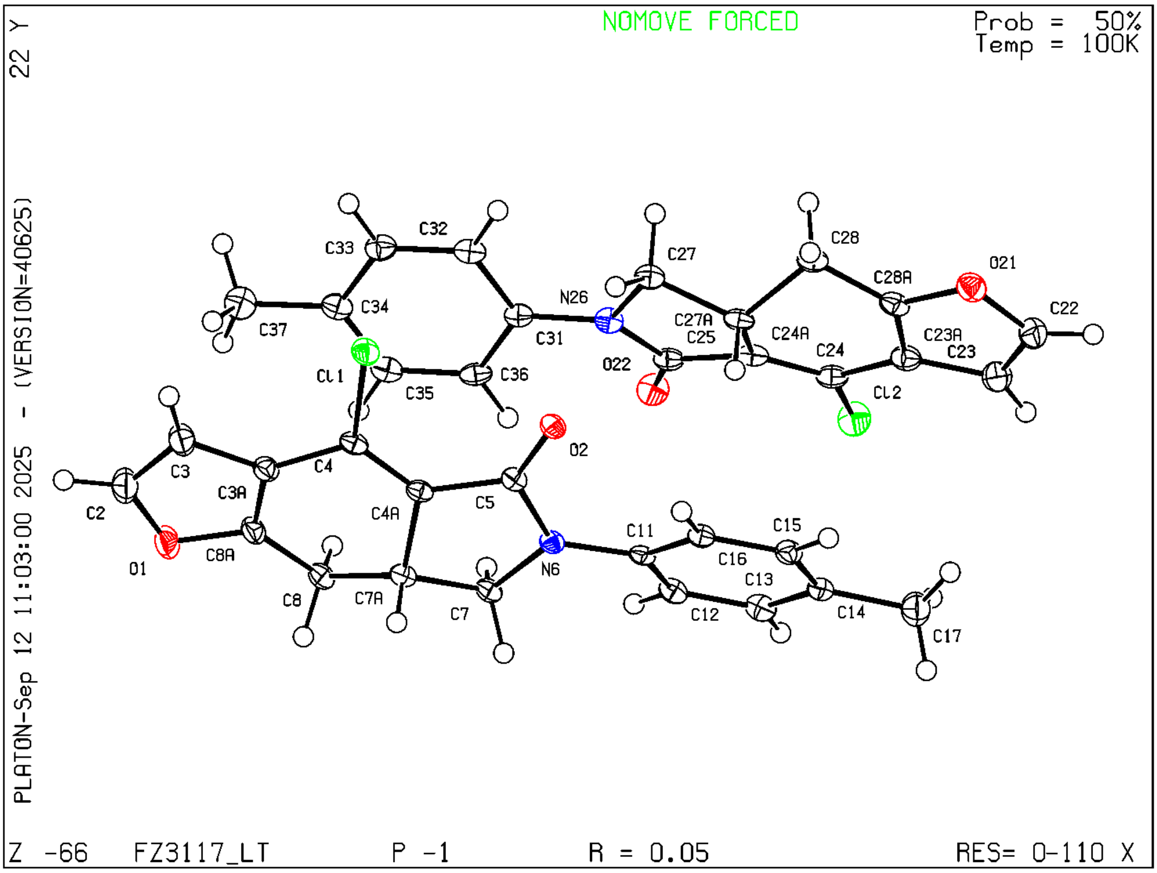

Supplement: Supplementary file 3 [file e-82-00212-sup3.pdf]
