# Supplementary material for: Synthesis and structure of (7aRS)-4-chloro-6-(4-methyl­phen­yl)-6,7,7a,8-tetra­hydro-5H-indeno­[5,6-b]furan-5-one, a fused-ring system arising from a new variant of the IMDAV reaction
Source: Acta Crystallogr E Crystallogr Commun. 2026 Jan 27;82(Pt 2):212–6. doi: 10.1107/S2056989026000629 (PMC12874248; doi:10.1107/S2056989026000629)
Supplement: Supplementary file 4 [file e-82-00212-sup4.pdf]

Synthesis, crystal structure and Hirshfeld surface analysis of (7aRS)-4-chloro-6-(4-methylphenyl)-6,7,7a,8-tetrahydro-5*H*-indeno [5,6-*b*]furan-5-one

Kseniia A. Alekseeva,[a] Atash V. Gurbanov,[b] Mikhail S. Grigoriev,[c] Victoria I. Salakhova,[a] Ekaterina A. Akishina,[d] Mohammed Hadi Al-Douh,[e]* Tuncer Hökelek[f] and Khudayar I. Hasanov[g]

[a]RUDN University, 6 Miklukho-Maklaya St., Moscow 117198, Russian Federation, [b]Excellence Center, Baku State University, Z. Khalilov Str. 33, AZ 1148, Baku, Azerbaijan, [c]Frumkin Institute of Physical Chemistry and Electrochemistry, Russian Academy of Sciences, Leninsky prosp. 31, Build. 4, Moscow 119071, Russian Federation, [d]Institute of Physical Organic Chemistry, National Academy of Sciences of Belarus, Surganov Str. 13, Minsk 220072, Belarus, [e]Chemistry Department, Faculty of Science, Hadhramout University, Mukalla, Hadhramout, Yemen, [f]Hacettepe University, Department of Physics, 06800 Beytepe-Ankara, Türkiye, and [g]Azerbaijan Medical University, Scientific Research Centre (SRC), A. Kasumzade Str. 14, AZ 1022, Baku, Azerbaijan
Correspondence email: m.aldouh@hu.edu.ye

Abstract

The asymmetric unit of the title compound, $C_{17}H_{14}ClNO_2$, (**1**) contains two crystallographically independent molecules, where the benzene and pyrrole rings are in screw-boat and half-chair conformations, respectively. In crystal, intermolecular C—H···O and C—H···Cl hydrogen bonds link the molecules into two dimensional networks, enclosing $R^3_3(19)$, $R^2_2(18)$ and $R^2_2(14)$ ring motifs. Further, the C—H···π(ring) interactions help to consolidate the packing. Hirshfeld surface analysis revealed that the most important contributions for the crystal packing are from H···H (41.4% and 41.5%), H···C/C···H (18.1% and 20.2%), H···O/O···H (16.0% and 13.4%) and H···Cl/Cl···H (13.4% and 11.9%) interactions.

1. Chemical context

Isoindole is one of the key heterocyclic scaffolds widely present in natural products, pharmaceuticals and materials (Heugebaert *et al.*, 2012; Bailly, 2023). Novel synthetic approaches to isoindole-based compounds, as well as their applications, continue to be reported regularly (Neto &Zeni, 2021; Hammouda & Elattar, 2022; Maharramov *et al.*, 2011; Ayoup *et al.*, 2023). We previously introduced a method for the synthesis of a fused isoindole framework *via* the intramolecular Diels-Alder reaction of vinylarenes (IMDAV strategy) (Krishna *et al.*, 2022; Voronov *et al.*, 2018). During this investaigation, it was found taht the IMDAV reaction of 3-(2-furyl)allylamines with bromomaleic anhydride proceeds with concominant dehydrobromination, affording the planar heterocyclic scaffold 5-oxo-4a,5,6,7,7a,8-hexahydro-4*H*-furo[2,3-*f*]isoindole (Alekseeva *et al.*, 2020; Pronina *et al.*, 2024). This observation prompted us to explore the reactivity of a broader range of 3-(aryl)allylamines with halogenated maleic anhydrides. In earlier studies, we demonstrated that the reaction of 3-(2-furyl)allylamine with dichloromaleic anhydride delivers the aromatic fuse isoinole derivative 6,7-dihydro-5*H*-furo[2,3-*f*]isoindol-5-one (Alekseeva *et al.*, 2025). By contrast, replacing dibromomaleic acid anhydride with dichloromaleic anhydride does not produce the analogues aromatic product. Although the reaction proceeds through the same sequence of elementary transformations, it terminates after decarboxylation and elimination. The resulting compound 4-chloro-6,7,7a,8-tetrahydro-5*H*-indeno[5,6-*b*]furan-5-one (**1**), is resistant to further oxidation under ambient conditions or in the presence of various oxidants (Fig. 1). Similarly to halogenated metal complexes (Gurbanov *et al.*, 2022; Shixaliyev *et al.*, 2013, 2014), the chlorine atom in the title compound can also act as the halogen bond donor in crystal packing diagram, leading to the supramolecular assembly. Herein, we have reported the synthesis, molecular and crystal structures together with the Hirshfeld surface analysis of the title compound (**1**).

2. Structural commentary

The asymmetric unit of the title compound (**1**) contains two crystallographically independent molecules (Fig. 2). In molecules (**a**) and (**b**), the planar, [*A* (O1/C2/C3/C3a/C8a) and *D* (C11–C16)] and [*E* (O21/C22/C23/C23*a*/C28*a*) and *H* (C31–C36)], rings are oriented at dihedral angles of [*A*/*D* = 23.26 (6)°] and [*E*/*H* = 14.62 (6)°]. Atoms C17 and C37 are −0.0286 (23) Å and 0.0351 (21) Å away from corresponding rings planes. On the other hand, the dihedral angles between the planar rings of the two molecules are *A*/*E* = 10.46 (7)°, *A*/*H* = 8.85 (7)°, *D*/*E* = 14.32 (6)° and *E*/*H* = 28.93 (6)°. The nonplanar, [*B* (C3a/C4/C4a/C7a/C8/C8a) and *F* (C23*a*/C24/C24*a*/C27*a*/C28/C28*a*)] and [*C* (N6/C4a/C5/C7/C7a) and *G* (N26/C24*a*/C25/C27/C27*a*)], rings are in screw-boat and half-chair conformations with the puckering parameters of [Q_T = 0.3713 (21) Å, θ = 117.15 (31)° and φ = 23.1 (4)° (for ring *B*)], [Q_T = 0.4054 (21) Å, θ = 63.89 (30)° and φ = 204.3 (3)° (for ring *F*)], [φ = 121.5 (5)° (for ring *C*)] and [φ = 308.0 (5)° (for ring *G*)], respectively (Figs. 3 a–d). There appear to not be any unusual bond distances or interbond angles in the molecules of the title compound (**1**).

3. Supramolecular features

In the crystal, intermolecular C—H···O and C—H···Cl hydrogen bonds (Table 2) link the molecules into two dimensional networks, enclosing $R^3_3(19)$, $R^2_2(18)$ and $R^2_2(14)$ ring motifs (Etter *et al.*, 1990) (Fig. 4). Further the C—H···π(ring) interactions (Table 2) help to consolidate the packing within the crystal.

4. Hirshfeld surface analysis

For visualizing the intermolecular interactions in the crystal of title compound (**1**), Hirshfeld surface (HS) analyses were carried out by using Crystal Explorer 17.5 (Spackman *et al.*, 2021). In the HSs plotted over d_{norm} (Figs. 5 *a* and *b*), the contact distances equal, shorter and longer with respect to the sum of van der Waals radii are shown by the white, red and blue colours, respectively. According to the two-dimensional fingerprint plots, H ··· H, H ··· C/C ··· H, H ··· O/O ··· H and H ··· Cl/Cl ··· H contacts make the most important contributions to the HSs (Tables 3 and 4, Figs. 6 and 7), and they have significant differences due to the different numbers and values of the close contacts.

5. Synthesis and crystallization

N-[(2*E*)-3-(Furan-2-yl)prop-2-en-1-yl]-4-methylaniline (0.28 g, 1.3 mmol) (**2**) was dissolved in dry CH_2Cl_2 (10 ml) and cooled to 251 K. Dichloromaleic anhydride (0.22 g, 1.3 mmol) was added, and the mixture was kept at 269 K for 9 d. The resulting precipitate was filtered, dissolved in AcOEt (10 ml), and stirred at 350 K for 30 min. Then, the precipitate was filtered off and washed with AcOEt (2 × 2 ml). The product was dried to constant weight to afford compound (**1**) as white solid (114.1 mg, 0.38 mmol, 29%, m.p. 425–428 K). A single-crystal suitable for X-ray analysis was obtained from DMSO-d_6 with heating to 353 K and following slow cooling to r.t. ^{1}H NMR (700.2 MHz, DMSO-d_6, 298 K) δ 7.72 (br. dd, J = 1.0, 1.9, 1H, H-2-furyl), 7.60 (d, J = 8.3, 2H, H-2,6-C_6H_4), 7.21 (d, J = 8.4, 2H, H-3,5-C_6H_4), 6.70 (br. d, J = 1.9, 1H, H-3-furyl), 4.04 (t, J = 8.8, 1H, H-7 A), 3.66 (dd, J = 7.9, 9.3, 1H, H-7B), 3.54–3.48 (m, 1H, H-7 A), 3.19 (dd, J = 9.3, 16.5, 1H, H-8 A), 2.82 (t, J = 16.7, 1H, H-8B), 2.28 (s, 3H, CH_3) p.p.m. ^{13}C NMR (176.1 MHz, DMSO-d_6, 298 K) δ 163.0 (C═O), 154.5, 143.8, 137.2, 133.5, 129.2 (2 C, C-2,6-C_6H_4), 124.6, 123.0, 120.0, 119.5, (2 C, C-3,5-C_6H_4), 107.4, 50.8, 35.1, 25.7, 20.5 p.p.m. IR (KBr), ν (cm^{-1}): 3102, 3045, 2840, 2602, 1742, 1694, 1514, 1414, 1253, 836. Anal. Calcd for $C_{17}H_{14}ClNO_2$: C, 68.12; H, 4.71; N, 4.67; Found: C 67.81; H 4.59; N 4.44.

6. Refinement

Crystal data, data collection and structure refinement details are summarized in Table 1. The C-bond hydrogen atom positions were calculated geometrically at distances of 1.00 (for methine CH), 0.95 (for aromatic CH), 0.99 (for methylene CH) and 0.98 Å (for CH_3) and refined using a riding model by applying the constraint of U_{iso} = k × U_{eq} (C),

where k = 1.5 for methyl H atoms and k = 1.2 for the other H atoms.

Table 1

Experimental details

Crystal data	
Chemical formula	$C_{17}H_{14}ClNO_2$
M_r	299.74
Crystal system, space group	Triclinic, $P\bar{1}$
Temperature (K)	100
a, *b*, *c* (Å)	9.4921 (7), 10.6159 (8), 15.1129 (11)
α, *β*, *γ* (°)	105.490 (3), 104.705 (3), 99.662 (3)
V (Å^3)	1373.39 (18)
Z	4
Radiation type	Mo *Kα*
μ (mm^{-1})	0.28
Crystal size (mm)	0.40 × 0.32 × 0.28
Data collection	
Diffractometer	Bruker *KAPPA APEX* II area-detector diffractometer
Absorption correction	Multi-scan<br>*SADABS* 2016/2: Krause, L., Herbst-Irmer, R., Sheldrick G.M. & Stalke D., J. Appl. Cryst. 48 (2015) 3-10
T_{min}, T_{max}	0.916, 1.000
No. of measured, independent and observed [$I > 2\sigma(I)$] reflections	24205, 7992, 5466
R_{int}	0.050
(sin $\theta/\lambda)_{max}$ (Å^{-1})	0.703
Refinement	
$R[F^2 > 2\sigma(F^2)]$, $wR(F^2)$, S	0.049, 0.114, 1.03
No. of reflections	7992
No. of parameters	381
H-atom treatment	H-atom parameters constrained
$\Delta\rho_{max}$, $\Delta\rho_{min}$ (e Å^{-3})	0.39, −0.38

Computer programs: *APEX3* (Bruker, 2018), *SAINT* (Bruker, 2018), *SHELXT* (Sheldrick, 2015a), *SHELXL2018*/3 (Sheldrick, 2015b), *ORTEP-3* for Windows (Farrugia, 2012), *WinGX* publication routines (Farrugia, 2012) and *PLATON* (Spek, 2009).

Table 2

Selected interatomic distances (Å)

I1···I2^{i}	3.9546 (2)	O8···H18^{ii}	2.45
I1···H10A^{ii}	3.16	O9···H12	2.62
Cl1···H15^{iii}	2.80	O10···H27C	2.35
O1···O10	2.623 (3)	O10···H23C^{vii}	2.68
O2···C4	3.098 (2)	O11···H10^{iv}	2.71
O3···C6	2.913 (4)	O11···H43^{vi}	2.60
O4···O1	3.134 (3)	O22···H8A^{viii}	2.51

O4···C3A	3.066 (3)
O4···O6^{iv}	3.146 (3)
O4···C1	2.798 (3)
O5···O11^{iv}	3.024 (6)
O5···O6^{iv}	2.639 (3)
O6···C36	2.894 (3)
O7···C28	2.817 (3)
O8···C30	2.980 (4)
O10···C27A	3.011 (3)
O10···C25	3.100 (4)
O1···H10O	1.87 (6)
O1···H49A^{iv}	2.65
O1···H50B	2.60
O2···H18	2.57
O2···H4B	2.51
O2···H30^{ii}	2.64
O3···H6	2.33
O3···H47B^{v}	2.65
O3···H22	2.70
O4···H50C^{iv}	2.63
O4···H42^{vi}	2.68
O4···H27B^{vi}	2.71
O4···H3C	2.44
O4···H49B	2.57
O5···H27B^{vi}	2.71
O5···H40^{vi}	2.64
O5···H11O^{iv}	2.1249
O6···H5O^{iv}	1.77 (6)
O6···H36	2.35
O7···H28A	2.71
O7···H3A^{vii}	2.51
O7···H42	2.66
O7···H28B	2.44
O8···H46	2.59
O8···H30	2.40
O8···H4B^{ii}	2.68
O22···H35^{viii}	2.46
C12···C43^{vi}	3.216 (4)
C12···C42^{vi}	3.238 (4)
C21···C22^{ix}	3.409 (3)
C21···C40^{vi}	3.387 (4)
C21···C35^{vi}	3.304 (4)
C1···H12	2.64
C4A···H10A	2.76
C7···H39^{vi}	2.87
C8···H39^{vi}	2.80
C10···H11O^{iv}	2.83
C16···H3A	2.82
C18···H27A^{vi}	2.89
C19···H27A^{vi}	2.83
C21···H22^{ix}	2.80
C24···H50C^{iv}	2.71
C24···H11O^{iv}	2.51
C24···H3C	2.64
C25···H5O^{iv}	2.81 (6)
C25···H36	2.72
C27···H40	2.57
C28A···H34A	2.80
C35···H34A^{x}	2.81
C40···H27A	2.84
C40···H34^{x}	2.81
C43···H37^{x}	2.73
H3A···H16	2.40
H10···H11O^{iv}	2.31
H10O···H12	2.35
H11O···H50C	2.27
H15···H19^{xi}	2.34
H21···H23B	2.35
H27B···H40	2.40
H28A···H42	2.21
H45···H47C	2.38

Symmetry codes: (i) $x-1, y-1, z-1$; (ii) $-x+1, -y, -z+1$; (iii) $-x+1, -y+2, -z+1$; (iv) $-x+1, -y+1, -z+1$; (v) $x-1, y, z+1$; (vi) $x-1, y, z$; (vii) $x+1, y, z$; (viii) $-x, -y+1, -z+1$; (ix) $-x, -y+1, -z+2$; (x) $-x+2, -y+1, -z+1$; (xi) $-x, -y, -z+1$.

Table 3

Hydrogen-bond geometry (Å, º)

Cg6 and Cg8 are the centroids of the (C11···C16) and (C31···C36), respectively.

D—H···*A*	*D*—H	H···*A*	*D*···*A*	*D*—H···*A*
C8—H8*A*···O22^{viii}	0.99	2.51	3.401 (2)	149
C15—H15···Cl1^{iii}	0.95	2.80	3.693 (2)	157
C27—H27*A*···O2	0.99	2.48	3.166 (2)	127
C35—H35···O22^{viii}	0.95	2.46	3.252 (2)	141
C28—H28*A*···*Cg*8^{iv}	0.99	2.72	3.581 (2)	146
C7*A*—H7*AA*···*Cg*6^{xii}	1.00	2.55	3.484 (2)	155

Symmetry codes: (iii) −*x*+1, −*y*+2, −*z*+1; (iv) −*x*+1, −*y*+1, −*z*+1; (viii) −*x*, −*y*+1, −*z*+1; (xii) −*x*, −*y*+2, −*z*+1.

Table 4

Comparison of the percentages for molecules **a** and **b**.

Contacts	**a**	**b**
H···H	41.4	41.5
H···C/C···H	18.1	20.2
H···O/O···H	16.0	13.4
H···Cl/Cl···H	13.4	11.9
C···C	4.4	6.0
C···O/O···C	1.9	1.9
C···Cl/Cl···C	1.9	1.9
O···Cl/Cl···O	1.2	1.5
H···N/N···H	0.9	1.1
C···N/N···C	0.3	0.3
N···N	0.2	0.1
O···O	0.2	0.0
N···O/O···N	0.2	0.2

Acknowledgements

Funding of this researh was provided by the Russian Science Foundation (Project No. 23–43-10024) and the Belarussian Republican Foundation for Fundemental Research (Project No. X23RNF-051). X-ray diifaction experiment was carried out at the Center of shared use of physical methods of investigation of IPCE RAS. This work was also supprted by the Baku State University and Azerbaijan Medical University. T. H. is also grateful to Hacettepe University Scientific Research Project Unit (Grant No. 013 D04 602 004). The author's contributions are as follows. Conceptualization, AVG and TH; synthesis, KAA and VIS; X-ray analysis, AVG, MSG and TH; Hirshfeld surface analysis, TH; founding, KIH; writing (review and editing of the manuscript) AVG, EAA and TH, supervision, TH and MHAD.

Funding information

References

Alekseeva, K. A., Grigoriev, M. S., Kolesnik, I. A., Hasanov, K. I., Nazarova, R. z., Akkurt, M. & Manaheloheh, G. M. (2025). *Acta Cryst.* E**81**, 844–848.

Alekseeva, K. A., Kvyatkovskaya, E. A., Nikitina, E. V., Zaytsev, V. P., Eroshkina, S. M., Shikhaliev, K. S., Truong, H. H., Khrustalev, V. N. & Zubkov, F. I. (2020). *Synlett* **31**, 255–260.

Ayoup, M. S., Mansour, A. F., Abdel-Hamid, H., Abu-Serie, M. M., Mohyeldin, K. T., Pomberio, A. J. & Lteleb, M. (2023). *Eur. J. Med. Chem.* **245**, 114865.

Baily, C. (2023). *Eur. J. Med. Chem. Rep.* **9**, 100112.

Bruker (2018). *APEX3* and *SAINT*. Bruker AXS, Madison, Wisconsin, USA.

Etter, M. C., MacDonald, J. C. & Bernstein, J. (1990). *Acta Cryst.* E**46**, 256–262.

Farrugia, L. J. (2012). *J. Appl. Cryst.* **45**, 849–854.

Gurbanov, A. V., Kuznetsov, M. L., Karmakar, A., Aliyeva, V. A., Mahmudov, K. T. & Pombeiro, A. J. L. (2022). *Dalton Trans.* **51**, 1019–1031.

Hammouda, M. M. & Elattar, K. M. (2022). *RSC Adv.* **12**, 24681–24712.

Heugebaert, T. S. A., Roman, B. I. & Stevens, C. V. (2012). *Chem. Soc. Rev.* **41**, 5626–5640.

Krause, L., Herbst-Irmer, R., Sheldrick, G. M. & Stalke, D. (2015). *J. Appl. Cryst.* **48**, 3–10.

Krishna, G., Grudinin, D. G., Nikitina, E. V. & Zubkov, F. I. (2022). *Synthesis* **54**, 797–863.

Maharramov, A. M., Khalilov, A. N., Sadikhova, N. D., Gurbanov, A. V. & Ng, S. W. (2011). *Acta Cryst.* E**67**, o1087.

Neto, J. S. S. & % Zeni, G. (2021). *Asian J. Org. Chem.* **10**, 1282–1318.

Pronina, A. A., Podrezova, A. G., Grigoriev, M. S., Hasanov, K. I., Sdikhova, N. D., Akkurt, M. & Bhattarai, A. (2024). *Acta Cryst.* E**80**, 777–782.

Sheldrick, G. M. (2015*a*). *Acta Cryst.* A**71**, 3–8.

Sheldrick, G. M. (2015*b*). *Acta Cryst.* C**71**, 3–8.

Shixaliyev, N. Q., Gurbanov, A. V., Maharramov, A. M., Mahmudov, K. T., Kopylevich, M. N., Martins, L. M. D. R. S., Muzalevskiy, V. M., Nenajdenko, V. G. & Pombeiro, A. J. L. (2014). *New J. Chem.* **38**, 4807–4815.

Shixaliyev, N. Q., Maharramov, A. M., Gurbanov, A. V., Nenajdenko, V. G., Muzalevskiy, V. M., Mahmudov, K. T. & Kopylevich, M. N. (2013). *Catal. Today* **217**, 76–79.

Spackman, P. R., Turner, M. J., McKinnon, J. J., Wolff, S. K., Grimwood, D. J., Jayatilaka, D. & Spackman, M. A. (2021). *J. Appl. Cryst.* **54**, 1006–1011.

Spek, A. L. (2009). *Acta Cryst.* D**65**, 148–155.

Voronov, A. A., Alekseeva, K. A., Ryzhkova, E. A., Zarubaev, V. V., Galochkina, A. V., Zaytsev, V. P., Majik, M. S., Tilve, S. G., Gurbanov, A. V. & Zubkov, F. I. (2018). *Tetrahedron Lett.* **59**, 1108–1111.

Figure 1

Reaction scheme for obtaining the title compound, **1**.

Figure 2

The asymmetric unit of the title compound **1** with atom-numbering scheme and 50% probability ellipsoids.

Figure 3

Conformations of (*a*) benzene (in **a**), (*b*) benzene (in **b**), (*c*) pyrrole (in **a**) and (*d*) pyrrole (in **b**) rings.

Figure 4

The partial packing diagram of the title compound **1**. Intermolecular C—H···O and C—H···Cl hydrogen bonds are shown as dashed lines. Nonbonding H atoms have been omitted for clarity.

Figure 5

Views of the three-dimensional Hirshfeld surfaces for molecules (*a*) **a** and (*b*) **b** plotted over d_{norm} in the ranges of −0.1611 to 1.3552 a.u. (for **a**) and −0.1810 to 1.2598 a.u. (for **b**).

Figure 6

The full two-dimensional fingerprint plots for molecule **a**, showing (*a*) all interactions, and delineated into (*b*) H ··· H, (*c*) H ··· C/C ··· H, (*d*) H ··· O/O ··· H, (*e*) H ··· Cl/Cl ··· H, (*f*) C ··· C, (*g*) C ··· O/O ··· C, (*h*) C ··· Cl/Cl ··· C, (i) O ··· Cl/Cl ··· O, (*j*) H ··· N/N ··· H, (*k*) C ··· N/N ··· C, (*l*) N ··· N, (*m*) O ··· O and (*n*) N ··· O/O ··· N interactions. The d_i and d_e values are the closest internal and external distances (in Å) from given points on the Hirshfeld surface contacts.

Figure 7

The full two-dimensional fingerprint plots for molecule **b**, showing (*a*) all interactions, and delineated into (*b*) H ··· H, (*c*) H ··· C/C ··· H, (*d*) H ··· O/O ··· H, (*e*) H ··· Cl/Cl ··· H, (*f*) C ··· C, (*g*) C ··· O/O ··· C, (*h*) C ··· Cl/Cl ··· C, (i) O ··· Cl/Cl ··· O, (*j*) H ··· N/N ··· H, (*k*) C ··· N/N ··· C, (*l*) N ··· O/O ··· N, (*m*) N ··· N and (*n*) O ··· O interactions. The d_i and d_e values are the closest internal and external distances (in Å) from given points on the Hirshfeld surface contacts.

supporting information

Synthesis, crystal structure and Hirshfeld surface analysis of (7aRS)-4-chloro-6-(4-methylphenyl)-6,7,7a,8-tetrahydro-5*H*-indeno [5,6-*b*]furan-5-one

Computing details

Data collection: *APEX3* (Bruker, 2018); cell refinement: *SAINT* (Bruker, 2018); data reduction: *SAINT* (Bruker, 2018); program(s) used to solve structure: *SHELXT* (Sheldrick, 2015a); program(s) used to refine structure: *SHELXL2018*/3 (Sheldrick, 2015b); molecular graphics: *ORTEP-3* for Windows (Farrugia, 2012); software used to prepare material for publication: *WinGX* publication routines (Farrugia, 2012) and *PLATON* (Spek, 2009).

(FZ3117_LT)

Crystal data

$C_{17}H_{14}ClNO_2$
M_r = 299.74
Triclinic, $P\bar{1}$
a = 9.4921 (7) Å
b = 10.6159 (8) Å
c = 15.1129 (11) Å
α = 105.490 (3)°
β = 104.705 (3)°
γ = 99.662 (3)°
V = 1373.39 (18) Å^3
Z = 4
F(000) = 624
D_x = 1.450 Mg m^{-3}
Mo $K\alpha$ radiation, λ = 0.71073 Å
Cell parameters from 3763 reflections
θ = 2.9–27.2°
μ = 0.28 mm^{-1}
T = 100 K
Bulk, colourless
0.40 × 0.32 × 0.28 mm

Data collection

Bruker KAPPA APEX II area-detector diffractometer
φ and ω scans
Absorption correction: multi-scan *SADABS* 2016/2: Krause, L., Herbst-Irmer, R., Sheldrick G.M. & Stalke D., J. Appl. Cryst. 48 (2015) 3-10
T_{min} = 0.916, T_{max} = 1.000
24205 measured reflections
7992 independent reflections
5466 reflections with $I > 2\sigma(I)$
R_{int} = 0.050
θ_{max} = 30.0°, θ_{min} = 4.1°
h = −13→13
k = −14→13
l = −21→21

Refinement

Refinement on F^2
Least-squares matrix: full
$R[F^2 > 2\sigma(F^2)]$ = 0.049
$wR(F^2)$ = 0.114
S = 1.03
7992 reflections
381 parameters
0 restraints
Hydrogen site location: inferred from neighbouring sites
H-atom parameters constrained
$w = 1/[\sigma^2(F_o^2) + (0.0459P)^2 + 0.1431P]$ where $P = (F_o^2 + 2F_c^2)/3$
$(\Delta/\sigma)_{max}$ = 0.001
$\Delta\rho_{max}$ = 0.39 e Å^{-3}
$\Delta\rho_{min}$ = −0.38 e Å^{-3}

Special details

Geometry. All e.s.d.'s (except the e.s.d. in the dihedral angle between two l.s. planes) are estimated using the full covariance matrix. The cell e.s.d.'s are taken into account individually in the estimation of e.s.d.'s in distances, angles and torsion angles; correlations between e.s.d.'s in cell parameters are only used when they are defined by crystal symmetry. An approximate (isotropic) treatment of cell e.s.d.'s is used for estimating e.s.d.'s involving l.s. planes.

Fractional atomic coordinates and isotropic or equivalent isotropic displacement parameters ($Å^2$)

	x	*y*	*z*	U_{iso}*/U_{eq}
Cl1	0.20099 (5)	0.71344 (4)	0.17374 (3)	0.01863 (11)
O1	−0.34679 (15)	0.54624 (13)	0.08878 (10)	0.0245 (3)
O2	0.31068 (14)	0.86345 (12)	0.39990 (9)	0.0160 (3)
N6	0.11862 (16)	0.84991 (13)	0.46830 (11)	0.0135 (3)
C2	−0.2972 (2)	0.5315 (2)	0.00927 (15)	0.0282 (5)
H2	−0.359754	0.487656	−0.055801	0.034*
C3	−0.1489 (2)	0.58738 (19)	0.03607 (14)	0.0231 (4)
H3	−0.088429	0.589974	−0.005073	0.028*
C3A	−0.1006 (2)	0.64226 (17)	0.13935 (13)	0.0161 (4)
C4	0.0447 (2)	0.70905 (16)	0.21219 (13)	0.0146 (4)
C4A	0.0489 (2)	0.76130 (16)	0.30398 (13)	0.0137 (4)
C5	0.1779 (2)	0.82930 (16)	0.39256 (13)	0.0132 (3)
C7	−0.0430 (2)	0.78547 (17)	0.43799 (13)	0.0153 (4)
H7A	−0.095954	0.844681	0.472261	0.018*
H7B	−0.060717	0.698063	0.450341	0.018*
C7A	−0.0948 (2)	0.76485 (17)	0.32964 (13)	0.0151 (4)
H7AA	−0.124127	0.848108	0.321139	0.018*
C8	−0.2285 (2)	0.64387 (18)	0.26781 (13)	0.0173 (4)
H8A	−0.219668	0.564803	0.289777	0.021*
H8B	−0.324336	0.665991	0.272153	0.021*
C8A	−0.2236 (2)	0.61474 (17)	0.16722 (14)	0.0176 (4)
C11	0.20179 (19)	0.91290 (16)	0.56732 (13)	0.0133 (3)
C12	0.1474 (2)	0.87824 (17)	0.63696 (13)	0.0163 (4)
H12	0.056975	0.809595	0.617625	0.020*
C13	0.2239 (2)	0.94284 (18)	0.73415 (14)	0.0189 (4)
H13	0.184339	0.918209	0.780647	0.023*
C14	0.3571 (2)	1.04281 (18)	0.76560 (13)	0.0178 (4)
C15	0.4116 (2)	1.07528 (17)	0.69497 (13)	0.0167 (4)
H15	0.503493	1.142248	0.714485	0.020*
C16	0.3360 (2)	1.01299 (16)	0.59750 (13)	0.0143 (4)
H16	0.375143	1.038167	0.551019	0.017*
C17	0.4378 (2)	1.1132 (2)	0.87198 (14)	0.0271 (5)
H17A	0.392484	1.186163	0.896273	0.041*
H17B	0.544309	1.150899	0.881880	0.041*
H17C	0.429041	1.048173	0.906968	0.041*
Cl2	0.45803 (6)	0.71875 (5)	0.81644 (3)	0.02434 (12)
O21	0.91589 (15)	0.99157 (12)	0.79973 (10)	0.0209 (3)
O22	0.26448 (15)	0.56575 (12)	0.59949 (9)	0.0213 (3)
N26	0.37337 (17)	0.61881 (14)	0.48907 (11)	0.0156 (3)
C22	0.9066 (2)	1.02191 (19)	0.89213 (15)	0.0233 (4)
H22	0.984685	1.081148	0.948176	0.028*
C23	0.7725 (2)	0.95676 (18)	0.89282 (14)	0.0226 (4)
H23	0.739609	0.960197	0.947583	0.027*
C23A	0.6898 (2)	0.88127 (18)	0.79382 (14)	0.0184 (4)
C24	0.5468 (2)	0.78260 (17)	0.74464 (14)	0.0171 (4)
C24A	0.4964 (2)	0.73906 (17)	0.64839 (14)	0.0161 (4)
C25	0.3642 (2)	0.63252 (17)	0.58064 (14)	0.0161 (4)
C27	0.5182 (2)	0.69567 (17)	0.49017 (14)	0.0164 (4)
H27A	0.503727	0.739177	0.439320	0.020*

H27B	0.586941	0.636469	0.480340	0.020*
C27A	0.5796 (2)	0.80141 (17)	0.59103 (13)	0.0152 (4)
H27C	0.546967	0.885038	0.586917	0.018*
C28	0.7511 (2)	0.84007 (17)	0.63645 (13)	0.0171 (4)
H28A	0.790695	0.758683	0.625005	0.020*
H28B	0.799184	0.902961	0.608444	0.020*
C28A	0.7813 (2)	0.90604 (17)	0.74133 (14)	0.0175 (4)
C31	0.2638 (2)	0.53323 (16)	0.40167 (13)	0.0146 (4)
C32	0.3051 (2)	0.49318 (17)	0.31801 (14)	0.0172 (4)
H32	0.407051	0.520776	0.320751	0.021*
C33	0.1978 (2)	0.41349 (17)	0.23128 (14)	0.0190 (4)
H33	0.227942	0.386828	0.175175	0.023*
C34	0.0470 (2)	0.37116 (17)	0.22377 (14)	0.0181 (4)
C35	0.0079 (2)	0.41115 (17)	0.30786 (14)	0.0179 (4)
H35	−0.094066	0.383242	0.305000	0.022*
C36	0.1134 (2)	0.49030 (17)	0.39536 (14)	0.0168 (4)
H36	0.083204	0.515619	0.451553	0.020*
C37	−0.0691 (2)	0.28754 (19)	0.12885 (14)	0.0245 (4)
H37A	−0.163967	0.254733	0.139807	0.037*
H37B	−0.034213	0.210403	0.098298	0.037*
H37C	−0.085093	0.342847	0.086424	0.037*

Atomic displacement parameters (Å^2)

	U^{11}	U^{22}	U^{33}	U^{12}	U^{13}	U^{23}
Cl1	0.0133 (2)	0.0242 (2)	0.0188 (2)	0.00305 (17)	0.00713 (19)	0.00671 (18)
O1	0.0135 (7)	0.0344 (8)	0.0186 (7)	0.0008 (6)	0.0019 (6)	0.0035 (6)
O2	0.0102 (6)	0.0176 (6)	0.0185 (7)	0.0018 (5)	0.0046 (5)	0.0042 (5)
N6	0.0102 (7)	0.0145 (7)	0.0159 (8)	0.0020 (6)	0.0053 (6)	0.0047 (6)
C2	0.0209 (11)	0.0404 (12)	0.0168 (11)	0.0036 (9)	0.0031 (9)	0.0039 (9)
C3	0.0171 (10)	0.0310 (10)	0.0180 (10)	0.0031 (8)	0.0036 (8)	0.0065 (8)
C3A	0.0132 (9)	0.0168 (8)	0.0179 (10)	0.0036 (7)	0.0044 (8)	0.0053 (7)
C4	0.0120 (9)	0.0141 (8)	0.0207 (10)	0.0038 (7)	0.0068 (8)	0.0083 (7)
C4A	0.0121 (9)	0.0118 (8)	0.0194 (9)	0.0039 (7)	0.0061 (8)	0.0067 (7)
C5	0.0129 (9)	0.0102 (8)	0.0174 (9)	0.0025 (7)	0.0045 (7)	0.0063 (7)
C7	0.0103 (9)	0.0171 (8)	0.0188 (10)	0.0025 (7)	0.0052 (8)	0.0064 (7)
C7A	0.0127 (9)	0.0140 (8)	0.0195 (10)	0.0043 (7)	0.0054 (8)	0.0060 (7)
C8	0.0101 (9)	0.0199 (9)	0.0203 (10)	0.0016 (7)	0.0043 (8)	0.0057 (7)
C8A	0.0114 (9)	0.0180 (9)	0.0193 (10)	0.0017 (7)	0.0011 (8)	0.0044 (7)
C11	0.0119 (9)	0.0117 (8)	0.0182 (9)	0.0058 (7)	0.0053 (7)	0.0057 (7)
C12	0.0133 (9)	0.0157 (8)	0.0221 (10)	0.0037 (7)	0.0061 (8)	0.0090 (7)
C13	0.0189 (10)	0.0236 (9)	0.0209 (10)	0.0082 (8)	0.0096 (8)	0.0128 (8)
C14	0.0165 (10)	0.0205 (9)	0.0174 (10)	0.0089 (7)	0.0052 (8)	0.0054 (7)
C15	0.0129 (9)	0.0151 (8)	0.0205 (10)	0.0026 (7)	0.0053 (8)	0.0039 (7)
C16	0.0138 (9)	0.0146 (8)	0.0173 (9)	0.0044 (7)	0.0069 (8)	0.0071 (7)
C17	0.0239 (11)	0.0332 (11)	0.0207 (11)	0.0055 (9)	0.0048 (9)	0.0061 (9)
Cl2	0.0249 (3)	0.0303 (3)	0.0217 (3)	0.0036 (2)	0.0120 (2)	0.0122 (2)
O21	0.0180 (7)	0.0202 (6)	0.0238 (8)	0.0042 (5)	0.0056 (6)	0.0072 (6)
O22	0.0213 (7)	0.0225 (7)	0.0229 (8)	0.0018 (6)	0.0112 (6)	0.0102 (6)
N26	0.0152 (8)	0.0150 (7)	0.0192 (8)	0.0032 (6)	0.0078 (7)	0.0079 (6)
C22	0.0243 (11)	0.0246 (10)	0.0193 (10)	0.0081 (8)	0.0036 (9)	0.0065 (8)
C23	0.0242 (11)	0.0249 (10)	0.0203 (10)	0.0085 (8)	0.0069 (9)	0.0086 (8)

C23A	0.0200 (10)	0.0185 (9)	0.0210 (10)	0.0085 (8)	0.0081 (8)	0.0094 (8)
C24	0.0180 (10)	0.0176 (9)	0.0224 (10)	0.0075 (7)	0.0122 (8)	0.0100 (8)
C24A	0.0166 (9)	0.0163 (8)	0.0221 (10)	0.0082 (7)	0.0107 (8)	0.0100 (7)
C25	0.0188 (10)	0.0158 (8)	0.0202 (10)	0.0087 (7)	0.0102 (8)	0.0099 (7)
C27	0.0149 (9)	0.0167 (8)	0.0216 (10)	0.0040 (7)	0.0098 (8)	0.0087 (7)
C27A	0.0150 (9)	0.0149 (8)	0.0217 (10)	0.0068 (7)	0.0097 (8)	0.0101 (7)
C28	0.0161 (9)	0.0154 (8)	0.0238 (10)	0.0050 (7)	0.0103 (8)	0.0082 (7)
C28A	0.0148 (9)	0.0149 (8)	0.0247 (10)	0.0058 (7)	0.0065 (8)	0.0082 (8)
C31	0.0169 (9)	0.0124 (8)	0.0188 (10)	0.0055 (7)	0.0083 (8)	0.0082 (7)
C32	0.0179 (10)	0.0163 (8)	0.0238 (10)	0.0066 (7)	0.0111 (8)	0.0108 (8)
C33	0.0222 (10)	0.0203 (9)	0.0202 (10)	0.0087 (8)	0.0109 (9)	0.0098 (8)
C34	0.0194 (10)	0.0148 (8)	0.0240 (10)	0.0065 (7)	0.0081 (8)	0.0101 (8)
C35	0.0168 (10)	0.0153 (8)	0.0260 (11)	0.0052 (7)	0.0095 (8)	0.0102 (8)
C36	0.0213 (10)	0.0134 (8)	0.0226 (10)	0.0085 (7)	0.0124 (8)	0.0093 (7)
C37	0.0239 (11)	0.0247 (10)	0.0250 (11)	0.0038 (8)	0.0088 (9)	0.0085 (8)

Geometric parameters (Å, º)

Cl1—C4	1.7236 (18)	Cl2—C24	1.7357 (17)
O1—C8A	1.361 (2)	O21—C28A	1.358 (2)
O1—C2	1.379 (2)	O21—C22	1.376 (2)
O2—C5	1.219 (2)	O22—C25	1.224 (2)
N6—C5	1.382 (2)	N26—C25	1.379 (2)
N6—C11	1.414 (2)	N26—C31	1.413 (2)
N6—C7	1.468 (2)	N26—C27	1.470 (2)
C2—C3	1.341 (3)	C22—C23	1.347 (3)
C2—H2	0.9500	C22—H22	0.9500
C3—C3A	1.431 (3)	C23—C23A	1.431 (3)
C3—H3	0.9500	C23—H23	0.9500
C3A—C8A	1.351 (2)	C23A—C28A	1.355 (2)
C3A—C4	1.449 (3)	C23A—C24	1.445 (3)
C4—C4A	1.337 (2)	C24—C24A	1.331 (3)
C4A—C5	1.473 (2)	C24A—C25	1.468 (3)
C4A—C7A	1.514 (2)	C24A—C27A	1.514 (2)
C7—C7A	1.528 (2)	C27—C27A	1.529 (3)
C7—H7A	0.9900	C27—H27A	0.9900
C7—H7B	0.9900	C27—H27B	0.9900
C7A—C8	1.531 (2)	C27A—C28	1.532 (2)
C7A—H7AA	1.0000	C27A—H27C	1.0000
C8—C8A	1.483 (3)	C28—C28A	1.481 (3)
C8—H8A	0.9900	C28—H28A	0.9900
C8—H8B	0.9900	C28—H28B	0.9900
C11—C12	1.389 (2)	C31—C36	1.394 (2)
C11—C16	1.397 (2)	C31—C32	1.398 (2)
C12—C13	1.381 (3)	C32—C33	1.382 (3)
C12—H12	0.9500	C32—H32	0.9500
C13—C14	1.388 (3)	C33—C34	1.392 (3)
C13—H13	0.9500	C33—H33	0.9500
C14—C15	1.394 (2)	C34—C35	1.392 (3)
C14—C17	1.507 (3)	C34—C37	1.500 (3)
C15—C16	1.381 (2)	C35—C36	1.382 (3)
C15—H15	0.9500	C35—H35	0.9500

C16—H16	0.9500	C36—H36	0.9500
C17—H17A	0.9800	C37—H37A	0.9800
C17—H17B	0.9800	C37—H37B	0.9800
C17—H17C	0.9800	C37—H37C	0.9800
I1···I2^{i}	3.9546 (2)	O8···H18^{ii}	2.45
I1···H10A^{ii}	3.16	O9···H12	2.62
Cl1···H15^{iii}	2.80	O10···H27C	2.35
O1···O10	2.623 (3)	O10···H23C^{vii}	2.68
O2···C4	3.098 (2)	O11···H10^{iv}	2.71
O3···C6	2.913 (4)	O11···H43^{vi}	2.60
O4···O1	3.134 (3)	O22···H8A^{viii}	2.51
O4···C3A	3.066 (3)	O22···H35^{viii}	2.46
O4···O6^{iv}	3.146 (3)	C12···C43^{vi}	3.216 (4)
O4···C1	2.798 (3)	C12···C42^{vi}	3.238 (4)
O5···O11^{iv}	3.024 (6)	C21···C22^{ix}	3.409 (3)
O5···O6^{iv}	2.639 (3)	C21···C40^{vi}	3.387 (4)
O6···C36	2.894 (3)	C21···C35^{vi}	3.304 (4)
O7···C28	2.817 (3)	C1···H12	2.64
O8···C30	2.980 (4)	C4A···H10A	2.76
O10···C27A	3.011 (3)	C7···H39^{vi}	2.87
O10···C25	3.100 (4)	C8···H39^{vi}	2.80
O1···H10O	1.87 (6)	C10···H11O^{iv}	2.83
O1···H49A^{iv}	2.65	C16···H3A	2.82
O1···H50B	2.60	C18···H27A^{vi}	2.89
O2···H18	2.57	C19···H27A^{vi}	2.83
O2···H4B	2.51	C21···H22^{ix}	2.80
O2···H30^{ii}	2.64	C24···H50C^{iv}	2.71
O3···H6	2.33	C24···H11O^{iv}	2.51
O3···H47B^{v}	2.65	C24···H3C	2.64
O3···H22	2.70	C25···H5O^{iv}	2.81 (6)
O4···H50C^{iv}	2.63	C25···H36	2.72
O4···H42^{vi}	2.68	C27···H40	2.57
O4···H27B^{vi}	2.71	C28A···H34A	2.80
O4···H3C	2.44	C35···H34A^{x}	2.81
O4···H49B	2.57	C40···H27A	2.84
O5···H27B^{vi}	2.71	C40···H34^{x}	2.81
O5···H40^{vi}	2.64	C43···H37^{x}	2.73
O5···H11O^{iv}	2.1249	H3A···H16	2.40
O6···H5O^{iv}	1.77 (6)	H10···H11O^{iv}	2.31
O6···H36	2.35	H10O···H12	2.35
O7···H28A	2.71	H11O···H50C	2.27
O7···H3A^{vii}	2.51	H15···H19^{xi}	2.34
O7···H42	2.66	H21···H23B	2.35
O7···H28B	2.44	H27B···H40	2.40
O8···H46	2.59	H28A···H42	2.21
O8···H30	2.40	H45···H47C	2.38
O8···H4B^{ii}	2.68		
C8A—O1—C2	105.98 (15)	C28A—O21—C22	105.81 (15)
C5—N6—C11	125.76 (15)	C25—N26—C31	125.57 (15)
C5—N6—C7	113.16 (15)	C25—N26—C27	112.78 (15)

C11—N6—C7	120.76 (14)	C31—N26—C27	121.47 (15)
C3—C2—O1	110.94 (18)	C23—C22—O21	111.23 (18)
C3—C2—H2	124.5	C23—C22—H22	124.4
O1—C2—H2	124.5	O21—C22—H22	124.4
C2—C3—C3A	105.88 (18)	C22—C23—C23A	105.63 (18)
C2—C3—H3	127.1	C22—C23—H23	127.2
C3A—C3—H3	127.1	C23A—C23—H23	127.2
C8A—C3A—C3	106.78 (17)	C28A—C23A—C23	106.58 (17)
C8A—C3A—C4	119.41 (17)	C28A—C23A—C24	118.40 (17)
C3—C3A—C4	133.72 (17)	C23—C23A—C24	134.71 (17)
C4A—C4—C3A	118.35 (16)	C24A—C24—C23A	119.11 (16)
C4A—C4—Cl1	124.56 (14)	C24A—C24—Cl2	123.94 (15)
C3A—C4—Cl1	117.09 (14)	C23A—C24—Cl2	116.76 (14)
C4—C4A—C5	130.45 (17)	C24—C24A—C25	130.58 (16)
C4—C4A—C7A	120.80 (16)	C24—C24A—C27A	120.66 (17)
C5—C4A—C7A	108.64 (15)	C25—C24A—C27A	108.76 (15)
O2—C5—N6	125.69 (17)	O22—C25—N26	125.33 (18)
O2—C5—C4A	128.31 (17)	O22—C25—C24A	127.97 (17)
N6—C5—C4A	105.99 (15)	N26—C25—C24A	106.69 (15)
N6—C7—C7A	103.42 (13)	N26—C27—C27A	103.70 (14)
N6—C7—H7A	111.1	N26—C27—H27A	111.0
C7A—C7—H7A	111.1	C27A—C27—H27A	111.0
N6—C7—H7B	111.1	N26—C27—H27B	111.0
C7A—C7—H7B	111.1	C27A—C27—H27B	111.0
H7A—C7—H7B	109.0	H27A—C27—H27B	109.0
C4A—C7A—C7	102.46 (14)	C24A—C27A—C27	102.94 (14)
C4A—C7A—C8	114.98 (15)	C24A—C27A—C28	113.40 (15)
C7—C7A—C8	115.92 (14)	C27—C27A—C28	115.40 (15)
C4A—C7A—H7AA	107.7	C24A—C27A—H27C	108.3
C7—C7A—H7AA	107.7	C27—C27A—H27C	108.3
C8—C7A—H7AA	107.7	C28—C27A—H27C	108.3
C8A—C8—C7A	105.98 (14)	C28A—C28—C27A	105.98 (15)
C8A—C8—H8A	110.5	C28A—C28—H28A	110.5
C7A—C8—H8A	110.5	C27A—C28—H28A	110.5
C8A—C8—H8B	110.5	C28A—C28—H28B	110.5
C7A—C8—H8B	110.5	C27A—C28—H28B	110.5
H8A—C8—H8B	108.7	H28A—C28—H28B	108.7
C3A—C8A—O1	110.42 (17)	C23A—C28A—O21	110.74 (17)
C3A—C8A—C8	126.87 (17)	C23A—C28A—C28	126.47 (17)
O1—C8A—C8	122.58 (16)	O21—C28A—C28	122.47 (16)
C12—C11—C16	118.72 (17)	C36—C31—C32	118.53 (18)
C12—C11—N6	119.70 (15)	C36—C31—N26	121.87 (16)
C16—C11—N6	121.55 (15)	C32—C31—N26	119.57 (16)
C13—C12—C11	120.48 (17)	C33—C32—C31	120.14 (17)
C13—C12—H12	119.8	C33—C32—H32	119.9
C11—C12—H12	119.8	C31—C32—H32	119.9
C12—C13—C14	121.67 (17)	C32—C33—C34	121.91 (18)
C12—C13—H13	119.2	C32—C33—H33	119.0
C14—C13—H13	119.2	C34—C33—H33	119.0
C13—C14—C15	117.27 (17)	C35—C34—C33	117.24 (18)
C13—C14—C17	120.81 (16)	C35—C34—C37	121.25 (17)
C15—C14—C17	121.92 (17)	C33—C34—C37	121.51 (18)

C16—C15—C14	121.95 (17)	C36—C35—C34	121.81 (18)
C16—C15—H15	119.0	C36—C35—H35	119.1
C14—C15—H15	119.0	C34—C35—H35	119.1
C15—C16—C11	119.90 (16)	C35—C36—C31	120.37 (17)
C15—C16—H16	120.1	C35—C36—H36	119.8
C11—C16—H16	120.1	C31—C36—H36	119.8
C14—C17—H17A	109.5	C34—C37—H37A	109.5
C14—C17—H17B	109.5	C34—C37—H37B	109.5
H17A—C17—H17B	109.5	H37A—C37—H37B	109.5
C14—C17—H17C	109.5	C34—C37—H37C	109.5
H17A—C17—H17C	109.5	H37A—C37—H37C	109.5
H17B—C17—H17C	109.5	H37B—C37—H37C	109.5

C8A—O1—C2—C3	0.4 (2)	C28A—O21—C22—C23	0.9 (2)
O1—C2—C3—C3A	−0.4 (2)	O21—C22—C23—C23A	−0.9 (2)
C2—C3—C3A—C8A	0.3 (2)	C22—C23—C23A—C28A	0.5 (2)
C2—C3—C3A—C4	176.56 (19)	C22—C23—C23A—C24	173.68 (19)
C8A—C3A—C4—C4A	−10.9 (2)	C28A—C23A—C24—C24A	−13.0 (3)
C3—C3A—C4—C4A	173.19 (18)	C23—C23A—C24—C24A	174.46 (19)
C8A—C3A—C4—Cl1	168.34 (13)	C28A—C23A—C24—Cl2	162.26 (14)
C3—C3A—C4—Cl1	−7.6 (3)	C23—C23A—C24—Cl2	−10.3 (3)
C3A—C4—C4A—C5	177.46 (15)	C23A—C24—C24A—C25	173.90 (17)
Cl1—C4—C4A—C5	−1.7 (3)	Cl2—C24—C24A—C25	−1.0 (3)
C3A—C4—C4A—C7A	−6.9 (2)	C23A—C24—C24A—C27A	−5.9 (2)
Cl1—C4—C4A—C7A	173.96 (12)	Cl2—C24—C24A—C27A	179.24 (13)
C11—N6—C5—O2	−0.7 (3)	C31—N26—C25—O22	5.3 (3)
C7—N6—C5—O2	−174.11 (15)	C27—N26—C25—O22	−169.96 (17)
C11—N6—C5—C4A	−179.74 (14)	C31—N26—C25—C24A	−175.69 (15)
C7—N6—C5—C4A	6.83 (17)	C27—N26—C25—C24A	9.10 (18)
C4—C4A—C5—O2	7.2 (3)	C24—C24A—C25—O22	5.5 (3)
C7A—C4A—C5—O2	−168.91 (16)	C27A—C24A—C25—O22	−174.75 (17)
C4—C4A—C5—N6	−173.78 (17)	C24—C24A—C25—N26	−173.57 (18)
C7A—C4A—C5—N6	10.12 (17)	C27A—C24A—C25—N26	6.23 (18)
C5—N6—C7—C7A	−20.48 (18)	C25—N26—C27—C27A	−20.27 (18)
C11—N6—C7—C7A	165.72 (13)	C31—N26—C27—C27A	164.30 (14)
C4—C4A—C7A—C7	161.67 (15)	C24—C24A—C27A—C27	161.95 (16)
C5—C4A—C7A—C7	−21.79 (16)	C25—C24A—C27A—C27	−17.86 (18)
C4—C4A—C7A—C8	35.0 (2)	C24—C24A—C27A—C28	36.6 (2)
C5—C4A—C7A—C8	−148.43 (14)	C25—C24A—C27A—C28	−143.25 (15)
N6—C7—C7A—C4A	24.41 (16)	N26—C27—C27A—C24A	21.99 (17)
N6—C7—C7A—C8	150.44 (14)	N26—C27—C27A—C28	146.07 (14)
C4A—C7A—C8—C8A	−40.77 (19)	C24A—C27A—C28—C28A	−43.86 (18)
C7—C7A—C8—C8A	−160.18 (15)	C27—C27A—C28—C28A	−162.26 (13)
C3—C3A—C8A—O1	−0.1 (2)	C23—C23A—C28A—O21	0.0 (2)
C4—C3A—C8A—O1	−176.96 (14)	C24—C23A—C28A—O21	−174.44 (14)
C3—C3A—C8A—C8	175.90 (17)	C23—C23A—C28A—C28	173.59 (16)
C4—C3A—C8A—C8	−1.0 (3)	C24—C23A—C28A—C28	−0.9 (3)
C2—O1—C8A—C3A	−0.2 (2)	C22—O21—C28A—C23A	−0.57 (19)
C2—O1—C8A—C8	−176.36 (17)	C22—O21—C28A—C28	−174.43 (15)
C7A—C8—C8A—C3A	26.7 (2)	C27A—C28—C28A—C23A	29.3 (2)
C7A—C8—C8A—O1	−157.82 (15)	C27A—C28—C28A—O21	−157.86 (15)
C5—N6—C11—C12	−152.02 (16)	C25—N26—C31—C36	23.6 (2)

C7—N6—C11—C12	20.9 (2)	C27—N26—C31—C36	−161.56 (15)
C5—N6—C11—C16	30.0 (2)	C25—N26—C31—C32	−158.39 (16)
C7—N6—C11—C16	−157.00 (15)	C27—N26—C31—C32	16.4 (2)
C16—C11—C12—C13	0.8 (2)	C36—C31—C32—C33	0.4 (2)
N6—C11—C12—C13	−177.24 (15)	N26—C31—C32—C33	−177.65 (14)
C11—C12—C13—C14	−0.6 (3)	C31—C32—C33—C34	0.3 (3)
C12—C13—C14—C15	−0.3 (3)	C32—C33—C34—C35	−0.7 (2)
C12—C13—C14—C17	179.27 (17)	C32—C33—C34—C37	178.48 (15)
C13—C14—C15—C16	1.1 (3)	C33—C34—C35—C36	0.4 (2)
C17—C14—C15—C16	−178.52 (17)	C37—C34—C35—C36	−178.76 (15)
C14—C15—C16—C11	−0.9 (3)	C34—C35—C36—C31	0.3 (2)
C12—C11—C16—C15	0.0 (2)	C32—C31—C36—C35	−0.7 (2)
N6—C11—C16—C15	177.92 (15)	N26—C31—C36—C35	177.33 (14)

Symmetry codes: (i) $x-1, y-1, z-1$; (ii) $-x+1, -y, -z+1$; (iii) $-x+1, -y+2, -z+1$; (iv) $-x+1, -y+1, -z+1$; (v) $x-1, y, z+1$; (vi) $x-1, y, z$; (vii) $x+1, y, z$; (viii) $-x, -y+1, -z+1$; (ix) $-x, -y+1, -z+2$; (x) $-x+2, -y+1, -z+1$; (xi) $-x, -y, -z+1$.

Hydrogen-bond geometry (Å, º)

Cg6 and Cg8 are the centroids of the (C11···C16) and (C31···C36), respectively.

D—H···*A*	*D*—H	H···*A*	*D*···*A*	*D*—H···*A*
C8—H8*A*···O22^{viii}	0.99	2.51	3.401 (2)	149
C15—H15···Cl1^{iii}	0.95	2.80	3.693 (2)	157
C27—H27*A*···O2	0.99	2.48	3.166 (2)	127
C35—H35···O22^{viii}	0.95	2.46	3.252 (2)	141
C28—H28*A*···*Cg*8^{iv}	0.99	2.72	3.581 (2)	146
C7*A*—H7*AA*···*Cg*6^{xii}	1.00	2.55	3.484 (2)	155

Symmetry codes: (iii) $-x+1, -y+2, -z+1$; (iv) $-x+1, -y+1, -z+1$; (viii) $-x, -y+1, -z+1$; (xii) $-x, -y+2, -z+1$.
